# Reassessing the Evolutionary Relationships of *Eriobotrya* and *Rhaphiolepis* (Rosaceae): Evidence from Micromorphology, Complete Nuclear Ribosomal DNA and Mitochondrial Genomic Data

**DOI:** 10.3390/biology14121740

**Published:** 2025-12-04

**Authors:** Muhammad Idrees, Zhiyong Zhang, Yunyun Lv, Meng Li, Hui Wang, Nan Zhang, Fajun Chen, Julian M. H. Shaw

**Affiliations:** 1 College of Life Science, Neijiang Normal University, Neijiang 641000, China; zhangzyong219@126.com (Z.Z.); lvyunyun_sci@foxmail.com (Y.L.); whscnj@126.com (H.W.); zhangnan@njtc.edu.cn (N.Z.); chenfj@njtc.edu.cn (F.C.); 2 Co-Innovation Center for Sustainable Forestry in Southern China, College of Biology and the Environment, Nanjing Forestry University, Nanjing 210037, China; limeng@njfu.edu.cn; 3 Cultivated Plant Diversity, Royal Horticultural Society, Wisely, Woking, Surrey GU23 6QB, UK; julianshaw@rhs.org.uk

**Keywords:** China, Maleae, nrDNA sequences, scanning electron microscopy, taxonomy

## Abstract

*Eriobotrya* and *Rhaphiolepis* are two closely related genera within the Maleae tribe of Rosaceae, and delineation of the boundary between these genera requires clarification. This study aims to reassess the phylogeny of two genera by integrating data from nuclear ribosomal DNA (nrDNA), mitochondrial DNA (mtDNA), and the micromorphological features of leaves, petioles, fruits, and fruit apical sepals from 53 accessions, including 16 nrDNA and one mtDNA sequences acquired from NCBI, representing 25 *Eriobotrya*, and 14 *Rhaphiolepis* species. Prior taxonomic investigations have often relied on either morphological or molecular methods; however, resolving the complex evolutionary background of these genera benefits from the application of molecular data with in-depth micromorphological analysis. Our findings indicate that molecular phylogeny derived from nrDNA sequences and leaf micromorphology elucidates the relationship between *Eriobotrya* and *Rhaphiolepis*, supporting the monophyly of *Rhaphiolepis* (with high support) and *Eriobotrya* (with moderate to low support). Supplementary micromorphological features (petioles, fruits, fruit apical sepals) support their classification as separate genera and aid in identification. Nevertheless, the mtDNA gene tree derived from 52 protein-coding genes offered restricted evolutionary insights due to low sequence variability, and displayed incongruence with the robust nuclear and morphological topologies. Furthermore, the mtDNA gene tree exhibited incongruent placements for four *Eriobotrya* species (*E. hookeriana*, *E. laoshanica*, *E. deflexa*, and *E. fragrans*) which clustered within the *Rhaphiolepis* clade, with support values ranging from low to high confidence. The observed topological incongruences, mito-nuclear discordance, and the congruent patterns of micromorphological and nrDNA sequences indicate a reticulate history. Further research employing whole genome sequencing may shed further light on the complex evolutionary history of this key clade.

## Simple Summary

This study aims to elucidate the phylogeny of two closely related Rosaceae genera, *Eriobotrya* and *Rhaphiolepis* inferred from integrated nuclear, mitochondrial and micromorphological methods with dense taxon sampling. *Eriobotrya* and *Rhaphiolepis* are classified as distinct, monophyletic genera based on congruent evidence from complete nuclear ribosomal DNA sequences (nrDNA) and a comprehensive analysis of micromorphological variables. Diagnostic features for distinguishing and classifying both *Eriobotrya* and *Rhaphiolepis* include epidermal cell shapes and anticlinal walls on both surfaces, cuticular folding, stomatal complexes and distribution, colleters, hydathodes, amphistomatic-type stomata located near the leaf margin, petiole surface sculpture and cross-section outline, fruit surface sculpture, and the outer and inner surfaces of the fruit apical sepals. These features are consistent with the well-supported phylogeny from nrDNA. The mtDNA gene tree exhibited a strong discordance signal, indicating a complex evolutionary history.

## 1. Introduction

**Figure 1:**
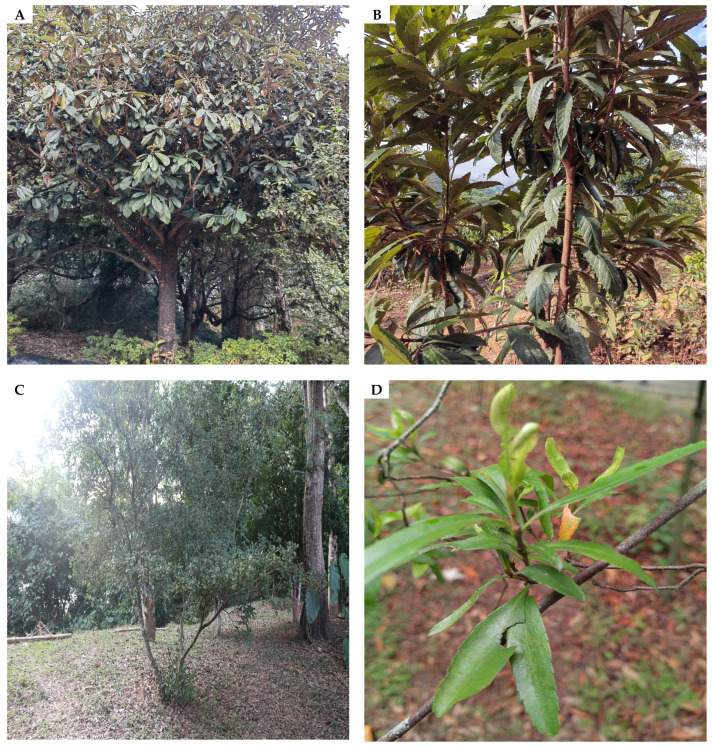
Gross morphology of *Eriobotrya* and *Rhaphiolepis*: (**A**) *Eriobotrya japonica*, (**B**) *E. deflexa*, (**C**) *Rhaphiolepis indica*, and (**D**) *R. salcifolia*.

Within the Maleae group of Rosaceae, *Eriobotrya* Lindl. [1] and *Rhaphiolepis* Lindl. [2] are two closely related genera (Figure 1), with approximately 36 species and 15 species recognized, respectively [3]. *Eriobotrya* species are evergreen, medium-sized trees characterized by the following: persistent stipules and apical sepals of the fruit; leaves exhibiting excurrent lateral veins; partially adnate, rectilinear, and paniculate inflorescences; fruit with reduced or absent sclereids; and three to five carpels (rarely two), which are free or up to one-third fused, with closed sutures—distinctive traits that distinguish *Eriobotrya* from other genera in Maleae [4,5,6,7,8,9,10]. The genus is prevalent in Eastern Asia (China, Taiwan), the East and West Himalayas (Bengal, Bhutan, Nepal, Assam and Sikkim), and Southeast Asia (especially Thailand, Indonesia, Myanmar, Vietnam, and Laos) [11,12,13,14]. Conversely, *Rhaphiolepis* species are evergreen shrubs or small trees, with caducous stipules and apical sepals of the fruit; leaves with curved lateral veins; inflorescences that are typically fully adnate, racemose, or rarely elongated paniculate; numerous scattered sclereids within the flesh of pomes; and generally two carpels (rarely three), fused at the base, with open sutures [4,5,6,7,8,9,10,15]. These species are extensively distributed in Eastern and Southeastern Asia, encompassing China (Taiwan, Hainan and Fujian), Japan, Central Malaya, northern Borneo, the Philippines, and Vietnam [16,17].

*Eriobotrya* and *Rhaphiolepis* have been treated as distinct genera based solely on morphology [1,2,4,7,11,18,19,20,21,22,23], and later molecular evidence [24,25,26,27,28,29,30,31,32,33,34,35,36,37], in the floristic literature [9,10,11,12,13,15,17,18,38]. The issue of generic identity emerged when Fay and Christenhusz [39] initially treated the genus *Eriobotrya* as a synonym of *Pyrus* L., and subsequently included it within *Rhaphiolepis* [40]. Nonetheless, recent research refutes the conclusions of Liu et al., and has resulted in gene trees that demonstrate *Eriobotrya* is distinct from *Rhaphiolepis*, exhibiting a robust monophyly [33,34,36,37]. The Plant of the World Online database [3] lists *Eriobotrya* as an accepted genus, while new species, including *E. tsiangii* [36], continue to be described in China, and other general and horticultural references continue to accept *Eriobotrya* [41,42,43].

In the Maleae tribe (Rosaceae), which includes several significant fruit crops and decorative plants (*Malus* Mill., *Sorbus* L., *Pyrus* L., *Rhaphiolepis*, and *Eriobotrya*), both intra- and inter-generic hybridization frequently occurs. This phenomenon aids in speciation and adaptation [26,44]. Approximately 54 million years ago (Ma), close to the Early Eocene Climatic Optimum, whole genome duplication (WGD) occurred in the most recent common ancestor of Maleae [37]. This agrees with the idea that Gillenieae (x = 9) served as a parental lineage for Maleae (x = 17). Rapid radiations, allopolyploid events, intergeneric hybridizations, gene copy loss after duplication, and ancient divergences among particular clades have historically made the genera in the tribe Maleae challenging to classify [7,37,44,45]. *Eriobotrya* experienced many ancient hybridization and chloroplast capture episodes on the Yunnan-Guizhou Plateau [40,46]. For a clearer taxonomic and systematic assessment, these two closely related genera (*Eriobotrya* and *Rhaphiolepis*) require further morphological and molecular information.

Several studies in the Rosaceae have demonstrated that examining the micromorphology of leaves and fruits with a scanning electron microscope (SEM) can provide new characters that help to identify and classify plants at the genus or species level. A few studies have examined the morphology of *Eriobotrya* and *Rhaphiolepis* species, including the whole plant [9,11,12,15,40,47], leaves [20], carpels [4], fruits/pome [6,48], flowers [49], pollens [21], and wood anatomy [50,51]. Although Zhang et al. [23], and Idrees et al. [52] provided a wide-ranging study of *Eriobotrya* using both qualitative and quantitative characters, other investigations focused on the leaf indumentum [53], leaf epidermis [54], leaf dimensions [55], leaf size, style count, and stamens [56], and fruit stone cells (sclereids) [22,57]; these studies lacked a micromorphological character analysis for both genera. Progress in this area necessitates the investigation of other features to gain a deeper understanding of the evolutionary traits and phylogenetic relationships between two closely related genera. These genera form a robustly supported monophyletic group within the tribe Maleae [25,26,34], excluding *Malus*, *Sorbus*, and *Pourthiaea* Decne. [37], a finding consistent with that of Robertson et al. [7] which also includes *Pyrus*. To our knowledge, no research has combined the micromorphological traits of *Eriobotrya* and *Rhaphiolepis* species in a phylogenetic framework.

There is a lack of consensus and clarity concerning the evolutionary relationships, taxonomy, and classification of the two genera. Many early methods for defining generic or species relationships and classifications depended on a limited number of species. To resolve the disputes regarding the taxonomy and phylogeny of *Eriobotrya* and *Rhaphiolepis*, we reconstructed the dated phylogeny utilizing expanded taxonomic sampling (including outgroups) and employing complete nuclear ribosomal DNA (nrDNA), mitochondrial DNA (mtDNA), and micromorphological characteristics to (1) evaluate the phylogenetic status of the genera *Eriobotrya* and *Rhaphiolepis*; (2) elucidate the micromorphological polymorphism, identifying the most informative features for distinguishing the two genera; and (3) offer an enhanced and stable taxonomic treatment of *Eriobotrya* and *Rhaphiolepis* that conveys optimal morphological and molecular information.

## 2. Materials and Methods

### 2.1. Plant Materials

In China, 53 accessions of *Eriobotrya* and *Rhaphiolepis* were collected, with 32 *Eriobotrya* accessions representing 25 species (including forms and unknown species) and 21 *Rhaphiolepis* accessions representing 14 species (including varieties). We obtained GenBank sequences for sixteen species of *Eriobotrya* and *Rhaphiolepis* for nuclear DNA study, as well as one species of *Eriobotrya* (*E. japonica*) for mitochondrial DNA. In addition, three species of *Phippsiomeles* B.B.Liu & J.Wen, and *Stranvaesia* Lindl. (nrDNA), and two species of *Malus* Mill., and *Pyrus* L. (mtDNA) were used as outgroups to help us comprehend their evolutionary relationships. Between 2016 and 2025, *Eriobotrya* and *Rhaphiolepis* species were collected from all around China through fieldwork or material exchange with botanical gardens. The obtained plant materials were stored at the College of Life Science Herbarium (NJTC). Tables S1–S3 contain details about the plant materials, as well as nrDNA and mtDNA sequence information.

### 2.2. Sequencing and Assembly

Fresh and silica-gel dried leaves were sent to Novogene Bioinformatics Technology Co., Ltd. (Beijing, China) for extraction of total genomic DNA for library preparation and Illumina sequencing. Sonication (Covaris LE220R-plus, Covaris USA, Woburn, MA, USA) was used to fragment the genomic DNA sample into 350 bp segments. Subsequently, DNA fragments were A-tailed, end-polished, and ligated using the full-length Illumina sequencing adapter, and further PCR amplification was performed. The Beckman Coulter, Beverly, MA, USA, AMPure XP equipment was used to purify the PCR products. After testing the library quality with the Agilent 5400 system (AATI), real-time PCR (1.5 nM) was used to quantify the results. More than 10 Gb of data were obtained for each sample after the qualifying libraries were combined and sequenced using the Illumina NovaSeq platform (Illumina, San Diego, CA, USA) with PE150 (Novogene, Beijing, China). Raw readings were cleaned and trimmed in paired-end mode using Fastp version 0.23.1 [58]. Reads shorter than 140 bp were eliminated (-l 140), as were sequence artifacts like adapter reads (>10 nucleotides aligned to the adapter, permitting ≤ 10% mismatches), unrecognizable bases (exceeding 10% uncertainty in either read), and reads with low quality bases (Phred quality < 5). To reconstruct mitochondrial genomic data, we first downloaded a set of conserved mitochondrial orthologous genes, and used Read2Tree v1.5.3 [59] to establish the reference framework. Paired-end reads were then mapped against these orthologs using minimap2 v2.30 [60], from which species-specific alignments were obtained. Read2Tree was subsequently employed to generate consensus sequences in phylogenetic alignment format across species. For the nuclear ribosomal DNA (nrDNA) region, consensus sequences were recovered using GetOrganelle v1.7.5.3 [61], which assembled the targeted region based on read mapping. Finally, progressiveMauve v2 [62] was applied to identify conserved blocks (regions shared across all sampled species) within the nrDNA alignments, providing reliable homologous sequences for phylogenetic analyses.

### 2.3. Phylogenetic Analysis

For phylogenetic inference, we first determined the best-fit substitution model using the ModelFinder module [63] implemented in IQ-TREE according to Bayesian Information Criteria (BIC), which were TIM3e + I + G4 (nrDNA) and TPM2u + F + I + G4 (mtDNA), respectively. Then, phylogenetic trees were reconstructed using IQ-TREE2 v2.2 [64], which combined maximum likelihood (ML) and Bayesian inference (BI) methods. The ML tree nodal support was assessed using SH-aLRT and 1000 ultrafast bootstrap repetitions, while the BI tree was inferred simultaneously to provide additional topological support. Figtree v1.4.5 [65] was then used to illustrate the tree.

#### Phylogenomic Discordance

Using the neighbor-net approach in SPLITSTREE v4, phylogenetic networks were constructed to investigate the evolutionary relationships within the lineages of both genera and to illustrate conflicts between gene trees [66]. This method has been widely used to display phylogenetic conflict and uncertainty resulting from possible reticulated evolution, as well as to identify species groups.

### 2.4. Morphological Evaluation

The micromorphologies of *Eriobotrya* and *Rhaphiolepis* species, encompassing adaxial and abaxial leaf surfaces, fruit surfaces, and the apical sepals of the fruits, were analyzed utilizing a light microscope and a scanning electron microscope (Thermo Scientific, Prisma, Shanghai, China) at Nanjing Forestry University. Fragments of leaves, fruits, and fruit apical sepals were dissected from the center, and surface pubescence was meticulously excised with fine tweezers under a stereomicroscope. To preserve surface morphology and prevent shrinkage, the samples underwent the following pretreatment. Specimens were preserved in 2.5% glutaraldehyde in a 0.1 M phosphate buffer (pH 7.2) at 4 °C for 6–8 h (or overnight for exceptionally thick samples); vacuum infiltration (two cycles of ~1–2 min of vacuum) was employed at the onset of fixation to improve reagent penetration. Subsequent to primary fixation, the samples were subjected to three rinses in the same solution (10 min each), and post-fixed in 1% osmium tetroxide (OsO_4_) in a 0.1 M phosphate buffer for 1 h (performed in a fume hood, protected from light). Following post-fixation, the specimens were rinsed once more (3 × 10 min) and dehydrated using a graded ethanol series: 30%, 50% (10 min each), 70%, 80%, 90%, 95% (15 min each), and 100% ethanol (two changes, 15 min each). Dehydrated samples were treated using a critical point dryer (CO_2_) following the manufacturer’s protocol to optimally preserve leaf surface and microstructure. Additionally, hexamethyldisilazane (HMDS) drying was employed as an alternative: samples were submerged in a 1:1 ethanol–HMDS solution for 10 min, then transferred to 100% HMDS for another 10 min. Subsequently, the samples were removed and air-dried in a fume hood for 30–60 min and then placed in a desiccator (or left overnight in a dry environment) to ensure full desiccation. The specimens were then affixed to aluminum stubs using double-sided conductive carbon tape and analyzed under a stereomicroscope to ensure integrity. The samples were sputter-coated with gold (Au) to a nominal thickness of 8–12 nm to ensure surface conductivity. SEM imaging was conducted at accelerating voltages of 15 kV for one to three minutes, with working distances of 8–15 mm.

The quantitative variables of the leaves were assessed using digital SEM photos processed with ImageJ v 1.54p software [67]. The adaxial surface included 7 qualitative variables including the epidermal cell shapes, the cuticular folding patterns, the anticlinal walls, the fine microrelief, the papillae, the base of trichomes, and the epicuticular wax deposits. The abaxial surface included 7 qualitative and 10 quantitative variables including the epidermal cell shapes, the anticlinal and periclinal walls, the base of fallen trichomes, the persistent non-glandular trichomes, the epicuticular wax deposits, the distribution of stomata, the primary and secondary stomata lengths and widths (µm), the outer ledge apertures of the primary and secondary stomata lengths and widths (µm), and stomatal density (stomatal number per 300 µm^2^). We additionally included 2 quantitative variables (leaf size and width) following the regional floras treatment, such as Flore du Cambodge, du Laos et du Vietnam [12], and Flora of China [9,15] (Table S4). The terminology about leaves was employed in accordance with the research of Song et al. [68,69] and Kumschova et al. [70].

To find significant differences across genera or species, one-way ANOVA was conducted in SPSS v26, and post hoc comparisons using the Least Significant Difference (LSD) were used to assume equal variance, interpreted at a significance level of *p* < 0.05. To ascertain the possible taxonomic diagnostic significance of various factors, cluster analysis was used to compare the leaf micromorphological characteristics of the two genera. We examined 16 qualitative and 10 quantitative variables in 32 accessions, treating each accession as an operational taxonomic unit (OTU) and assigning a taxonomic code (Table 1 and Table S4), following the process of Zhang et al. [23] without data standardization. Furthermore, we also evaluated the 9 stomatal variables separately, and data were standardized using SPSS software. Then, using the program PAST v.5 [71], the matrix was submitted to cluster analysis utilizing the Gower’s distance (for 16 qualitative and 10 quantitative variables), Euclidean distance (for stomatal variables), and UPGMA algorithm [23,72]. Principal Component Analysis (PCA) was performed using 26 variables, as indicated in Table 1 and Table S4, while 9 quantitative characteristics (stomatal variables) were used from the original data, following the approaches of Greenacre et al. [73] and Idrees et al. [52]. All multivariate analyses were conducted using PAST v.5 [71].

## 3. Results

### 3.1. Phylogenetic Analysis

#### 3.1.1. Taxon and Sequence Characteristics

The dataset used in this investigation comprised 53 accessions in total: 1 mtDNA and 16 nrDNA sequences of *Eriobotrya* and *Rhaphiolepis* species acquired from NCBI, with 17 newly added nrDNA and 38 mtDNA sequences from both genera. From the annotated mitochondrial genomes of the two genera, we identified 52 orthologous gene sets, with *sdh4*, *nad1*, *nad7*, *rps12*, *rnaseH*, and *mttB* genes having high nucleotide diversity, while *ORF300*, *ORF369* and *matR* genes had high GC contents (Table S5). Multiple sequence alignments of these orthologs produced a concatenated dataset of 42,440 aligned matrices containing 349 parsimony-informative (PI) sites and 592 variable sites, with total GC contents of 43.5%, which served as the basis for subsequent mtDNA analyses. In comparison, the nrDNA dataset produced 39,394 aligned matrices containing 1079 parsimony-informative (PI) sites and 2031 variable sites, with 55% total GC contents. Table 2 shows details on the recently identified sequences.

#### 3.1.2. Phylogenetic Analysis Based on Complete nrDNA Sequences

**Figure 2:**
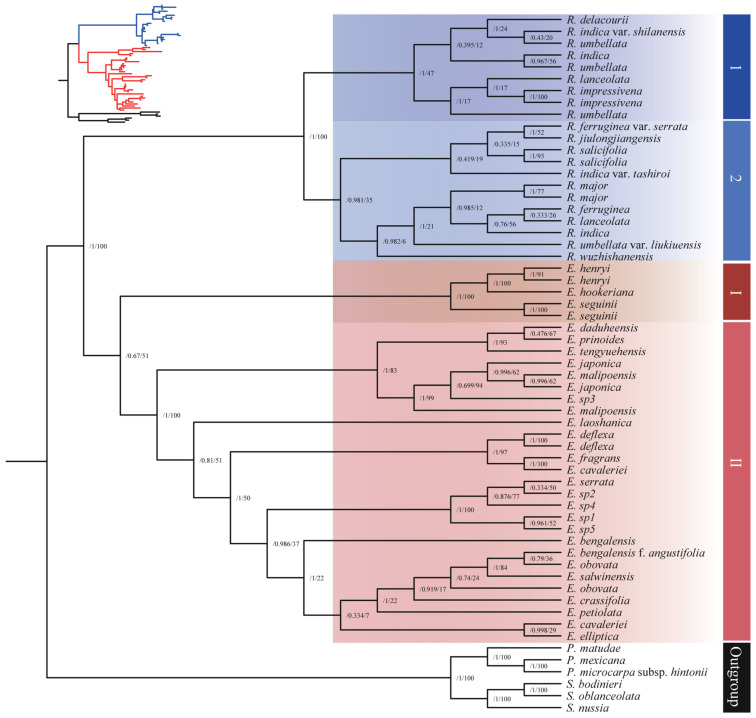
Phylogenetic tree of 53 accessions of *Eriobotrya* and *Rhaphiolepis* based on nrDNA sequences using both maximum likelihood (ML) and Bayesian inference (BI). The numbers at each node are the maximum likelihood bootstrap support/Bayesian posterior probabilities. The tree was rooted using the nrDNA sequences of *Phippsiomeles* and *Stranvaesia* species as outgroups.

The nrDNA matrix comprises 53 accessions, including 25 *Eriobotrya* species, 14 *Rhaphiolepis* species, and 6 additional species which serve as an outgroup. Our findings indicate that the complete nrDNA sequences elucidated the phylogenetic relationships between two closely related genera, affirming that *Rhaphiolepis* is monophyletic with high support (ML: 100% and BI: 1.0 posterior probability, respectively), while *Eriobotrya* is low to moderately supported (ML: 51% and BI: posterior probability 0.67, respectively) as a monophyletic group (Figure 2). This may be due to the uncertain phylogenetic positions of three *Eriobotrya* species (*E. henryi*, *E. hookeriana*, and *E. seguini*). The nrDNA phylogenetic tree identified two prominent clades within *Eriobotrya* and two within *Rhaphiolepis*. Clade I of *Eriobotrya* contained five accessions representing three species: two from China, *E. henryi* (from GenBank), and *E. seguinii* (from GenBank), and one from Bhutan, *E. hookeriana*, all of which had strong support values (ML: 91–100% and BI: posterior probability 1.0, respectively). Clade II is separated further into two subclades: A and B. Subclade A had eight accessions representing six species: *E. × daduheensis*, *E. prinoides*, *E. japonica*, *E. japonica* (from GenBank), *E. malipoensis*, *E. malipoensis* (from GenBank), *E. tengyuehensis*, and *E. sp3*, with low to moderate bootstrap values (ML: 62–99% and BI: posterior probability 0.47–1.0, respectively). Subclade B comprised 19 accessions representing 16 species: *E. laoshanica*, *E. deflexa*, *E. deflexa* (from GenBank), *E. cavaleriei*, *E. cavaleriei* (from GenBank), *E. fragrans*, *E. serrata*, *E. bengalensis*, *E. bengalensis* f. *angustifolia*, *E. salwinensis* (from GenBank), *E. obovata*, *E. obovata* (from GenBank), *E. crassifolia*, *E. elliptica*, *E. petiolata*, and *E. sp1* to *E. sp5*, exhibiting low to moderate support values. *E. deflexa*, *E. deflexa* (from GenBank), *E. cavaleriei*, *E. cavaleriei* (from GenBank), and *E. fragrans* exhibited high bootstrap values (91–100%).

The ML tree separated *Rhaphiolepis* species into two major clusters, 1 and 2 (Figure 2). Clade 1 includes nine accessions representing six species, *R. indica*, *R. indica* var. *shilanensis*, *R. × delacourii*, *R. umbellata1*, *R. umbellata2*, *R. umbellata* (from GenBank), *R. impressivena*, *R. impressivena* (from GenBank), and *R. lanceolata*, with low support values. Clade 2 is further subdivided into two subclades. Subclade I comprised five accessions representing four species, *R. ferrugenia* var. *serrata*, *R. jiulongjiangensis*, *R. salicifolia*, and *R. indica* var. *tashiroi*, with an additional *R. salicifolia* accession from GenBank. Subclade II encompassed seven accessions representing four species, *R. major*, *R. umbelatta* var. *liukiuensis*, *E. wuzhishanensis*, and four species from GenBank including *R. major*, *R. ferruginea*, *R. indica*, and *R. lanceolata*, with low bootstrap values.

#### 3.1.3. Phylogenetic Analysis Based on mtDNA Sequences

**Figure 3:**
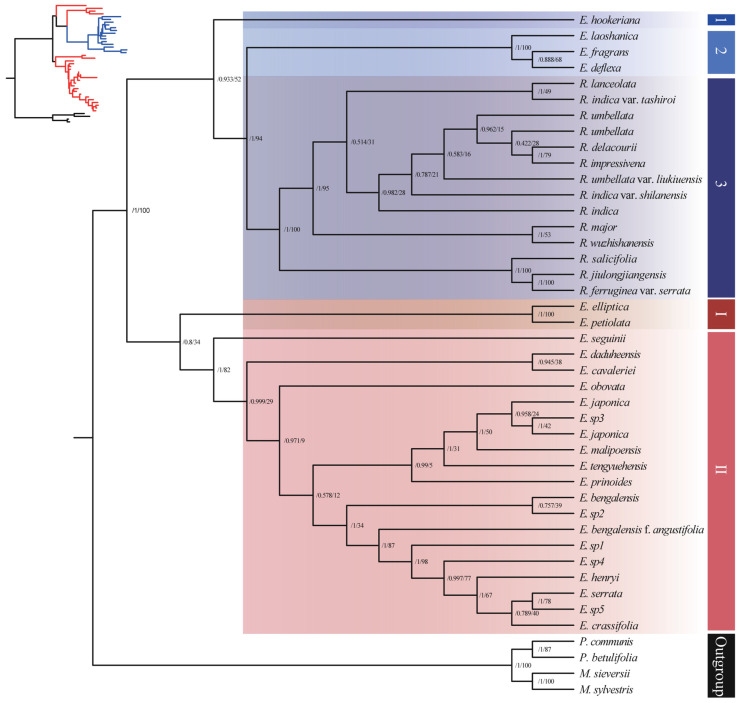
Phylogenetic tree of 39 accessions of *Eriobotrya* and *Rhaphiolepis* based on mtDNA sequences using both maximum likelihood (ML) and Bayesian inference (BI). The numbers at each node are the maximum likelihood bootstrap support/Bayesian posterior probabilities. The tree was rooted using the nrDNA sequences of *Pyrus* and *Malus* species as outgroups.

The mtDNA matrix based on 52 protein coding genes contained 43 accessions, representing 25 *Eriobotrya* species, 13 *Rhaphiolepis* species, and 4 of them were outgroups. The mtDNA sequences gene tree showed a strong discordance signal (Figure 3), with four distinct species of *Eriobotrya* (*E. hookeriana*, *E. laoshanica*, *E. deflexa*, and *E. fragrans*) forming a sister group within the *Rhaphiolepis* clade, with support values ranging from low to high (ML: 52–94% and BI: posterior probability 0.93–1.0, respectively). The relationships among the major subclades of *Eriobotrya* exhibited low support (ML: 34% and BI: posterior probability 0.8, respectively), while relationships within the *Rhaphiolepis* subclades showed high support values (ML: 100% and BI: posterior probability 1.0, respectively). This showed a complex hybridization from *Rhaphiolepis* into *Eriobotrya* species, actual biological conflict among loci, or a lack of informative characters. These results further support the previously published phylogeny based on nuclear and chloroplast genomes which highlights phylogenetic incongruence within the *Eriobotrya*–*Rhaphiolepis* complex [40].

#### 3.1.4. Network Analysis

The generated networks indicated both inter- and intrageneric reticulations in *Eriobotrya* and *Rhaphiolepis* (Figure S1a). The four complex *Eriobotrya* species, *E. deflexa*, *E. fragrans*, *E. laoshanica*, and *E. hookeriana*, despite having discordant phylogenetic positions in the distinct gene trees, formed complex networks, implying that multiple hybridization events took place among these species. Furthermore, the data demonstrated that these species, with *E. petiolata* and *E. elliptica*, are hybrid bridges; their presence increases network complexity, implying that there may be incomplete lineage sorting due to recent divergence from a common ancestor shared with *Rhaphiolepis* or ancient hybridization (ongoing introgression) from *Rhaphiolepis* into *Eriobotrya*. When the complex species were eliminated, there was no reticulation, confirming that they are causing the phylogenetic conflict (Figure S1b).

### 3.2. Morphological Analysis

#### 3.2.1. Adaxial Surface of the Leaf

**Figure 4:**
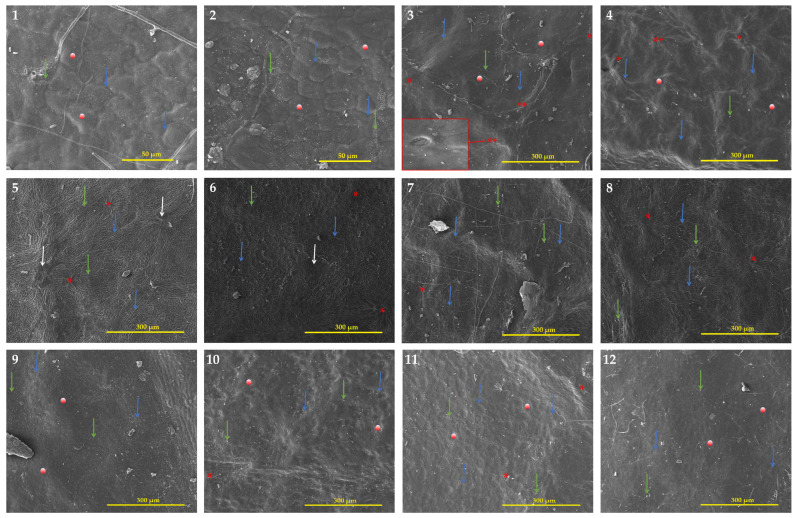
SEM micrographs of the adaxial surface of the leaves of *Eriobotrya* and *Rhaphiolepis* species: (**1**) *E. cavaleriei*, (**2**) *E. grandiflora*, (**3**) *E. japonica*, (**4**) *E. malipoensis*, (**5**) *E. bengalensis* f. *angustifolia*, (**6**) *E. deflexa*, (**7**) *E. elliptica*, (**8**) *E. tengyuehensis*, (**9**) *R. × delacourii*, (**10**) *R. integerrima*, (**11**) *R. indica*, and (**12**) *R. lanceolata*. Designations: The blue arrows are anticlinal walls, the green arrows are wax deposits, the white arrows are papillae formations, the white-red circles are periclinal walls, a single asterisk indicates the base of fallen trichomes, and double asterisks indicates stomata. Scale bars: 50–300 µm.

Table 3 provides information on the quantitative and qualitative characteristics of the leaf epidermis, whereas Figure 4 showed selected species SEM images and Figures S2–S8 showed all species within both genera. Regarding the adaxial epidermal cell shape of *Eriobotrya* and *Rhaphiolepis*, three groups were recognized: irregular (Group 1), and polygonal (Group 2) within *Eriobotrya* species, and irregular-polygonal or thin-verrucous (Group 3) in *Rhaphiolepis* species. On the investigated species epidermal surface, a folded microrelief with a distinct arrangement was discovered. Three distinct kinds were noted: long, straight-curve folds that covered the entire surface; small, straight or undulate folds; and smooth cuticular folds with several flat areas in between. Due to the numerous folds that were situated beside dense parallel cuticular folds and grooves, for most *Eriobotrya* species, the anticlinal walls of the epidermal cells were not discernible in the SEM. The cuticular folds formed papillae formations with folded microrelief on the epidermal cell surface of both genera. A mature papilla is composed of two structural components or parts: a leg with strong radially diverging ribs and a head that compacts the expanded top. In most cases, the ribs of the cell leg are joined to the microtension bar or to comparable formations of the nearby cells. Some cells were classified as sparse because they lacked or had diminished papillary structures. In both genera, the pavement cells’ anticlinal walls created patterns that were either raised or depressed, with the periclinal walls’ flat surfaces or ridge-convex striated surfaces being highlighted. The adaxial surface of the leaves was covered in simple multi-cellular trichomes, which mainly fell during the early stages of development, or remained persistent throughout. Two *Eriobotrya* species (*E. bengalensis* and *E. condaoensis*), and one *Rhaphiolepis* (*R. jiulongjiangensis*) are completely glabrous in life, as evidenced by the absence of a trichome base. In *Eriobotrya*, a rosette of four to six cells rose above the pavement cells, encircling the base of the descending trichomes with parallel striate ornamentation, whereas in *Rhaphiolepis*, the base of the fallen trichomes was tubular and circular above the cells. Epicuticular wax deposits were detected in both genera, and four forms of wax were identified: granule, sparsely granule, rodlet, and sparsely rodlet. Granules are identified as irregular, mainly amorphous, spherical crystalloids on the surface, whereas rodlets are identified as slender needle-shaped waxes that are primarily narrow and create elongated threads on the surface. In all the tested species, a combination of the aforementioned wax types was found on the surface of the inspected specimens, hence wax type cannot be utilized to distinguish between the two genera.

**Figure 5:**
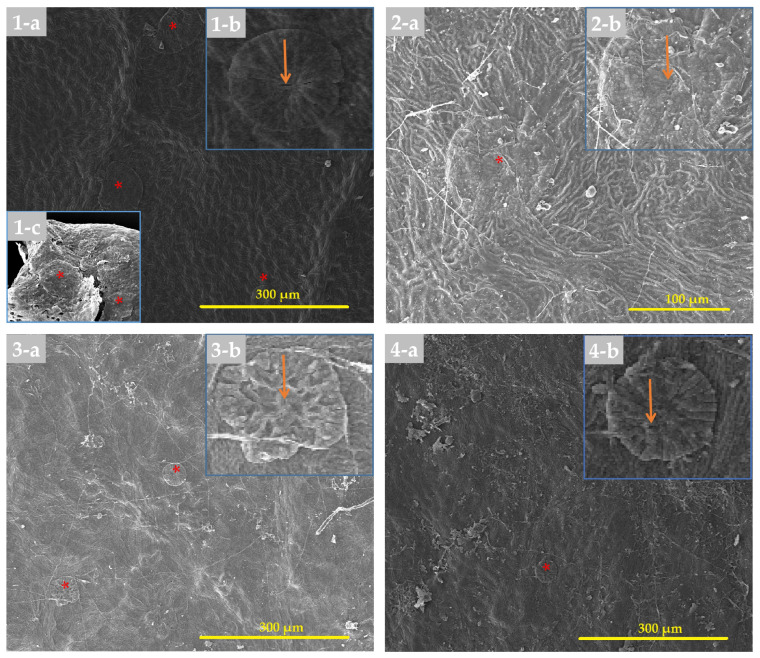
Laminar hydathodes on the adaxial leaf surface of *Eriobotrya*, using SEM micrography: (**1-a**–**1-c**) *E. fragrans*, (**2-a**–**2-b**) *E. obovata*, (**3-a**–**3-b**) *E. unknown* sample, and (**4-a**–**4-b**) *E. serrata*. Designations: The orange arrows indicate open pores, and a single asterisk indicates hydathodes. Scale bars: 100–300 µm.

The structure of laminar hydathodes in the studied *Eriobotrya* species was largely preserved. All species possessed epithemal hydathodes, consisting of three primary components: water holes, epithems, and tracheids. The water hole comprised open or slightly recessed pairs of guard cells, partially concealed by subsidiary cells. The epithem appeared as a group of tiny achlorophyllous cells beneath the water pores. An improperly organized network of xylem tracheids provided water to the epithelium and connected it to the leaf apex. The same structure was observed in *Eriobotrya* species, including *E. fragrans*, *E. salwinensis*, *E. obovata*, and an unknown sample from Yunnan, China (Figure 5), but not in any *Rhaphiolepis* species. Hydathode distribution (laminar, marginal, and apical) varied by species, although they were always more common, if not exclusively, on the surface most of those exposed to the atmosphere. In addition, another undiscovered volcano-like structure was discovered in two *Eriobotrya* species, *E. condaoensis* and *E. henryi*, with a rounded apex, raphides, and a sunken pore.

**Figure 6:**
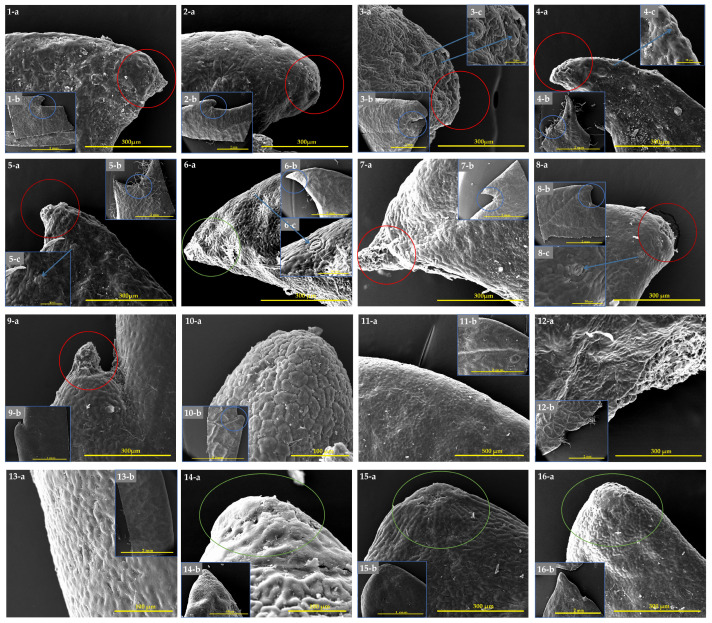
Colleters in *Eriobotrya* and *Rhaphiolepis* associated with the leaf margin apex (**1**–**16-a**), in sinuses between leaf margin apex (**1**–**16-b**), with surrounding stomata (**3**–**6c**), and colleters on the leaf apex (**6-a**,**b**, **14**–**16-a**,**b**): (**1-a**–**1-b**) *E. bengalensis*, (**2-a**–**2-b**) *E. bengalensis* f. *angustifolia*, (**3-a**–**3-c**) *E. crassifolia*, (**4-a**–**4-c**) *E. malipoensis*, (**5-a**–**5-c**) *E. deflexa*, (**6-a**–**6-b**) *E. deflexa* leaf apex, (**7-a**–**7-b**) *E. henryi*, (**8-a**–**8-c**) *E. prinoides*, (**9-a**–**9-b**) *R. lanceolata*, (**10-a**–**10-b**) *R. jiulongjiangensis*, (**11-a**–**11-b**) *R. umbellata*, (**12-a**–**12-b**) *R. wuzhishanensis*, (**13-a**–**13-b**) *R. × delacourii*, (**14-a**–**14-b**) *R. indica* leaf apex, (**15-a**–**15-b**) *R. lanceolata* leaf apex, and (**16-a**–**16-b**) *R. salicifolia* leaf apex. Designations: The red circles are colleters in the leaf margin, the blue circles are colleters in the sinuses between the leaf margin; the green circles indicate colleters in the leaf apex, and the blue arrows indicate stomata. Scale bars: 100–500 µm and 1–2 mm.

Leaf margin colleters have a short stalk with two to three rows of cells and a multi-cellular head. As a colleter’s head dries out over time, an abscission zone forms in the stalk. Under certain circumstances, secretion continues nearly until the leaves completely separate. Droplets of the secretion are released at the head and then spread out over the surface of the leaf tooth. Colleters are categorized in the literature as marginal, located at the apices of the teeth, and apical, ending the main vein (Figure 6). Spherical colleter heads have been discovered in the sinuses between the leaf margin apex, and the secretory head can be either elongated or triangular. *Eriobotrya* (Figure 6: nos. 1–8) had secretory “colleters” at the leaf teeth apex and in the sinuses between the leaf teeth. The adaxial surface of the leaf had many anomocytic stomata, primarily on the surface close to the margin apex, while colleters were found at the leaf margin apex in *R. lanceolata* (Figure 6: no. 9), in sinuses between the leaf teeth in *R. jiulongjiangensis* (Figure 6: no. 10), and on the adaxial surface of either the leaf apex or the margin apex of *R. indica*, *R. lanceolata*, and *R. salicifolia* (Figure 6: nos. 14–16), without any surrounding anomocytic stomata. We were unable to observe the colleters in most *Rhaphiolepis* species because the leaf margin and apex were completly entire (Figure 6: nos. 11–13).

#### 3.2.2. Abaxial Surface of the Leaf

**Figure 7:**
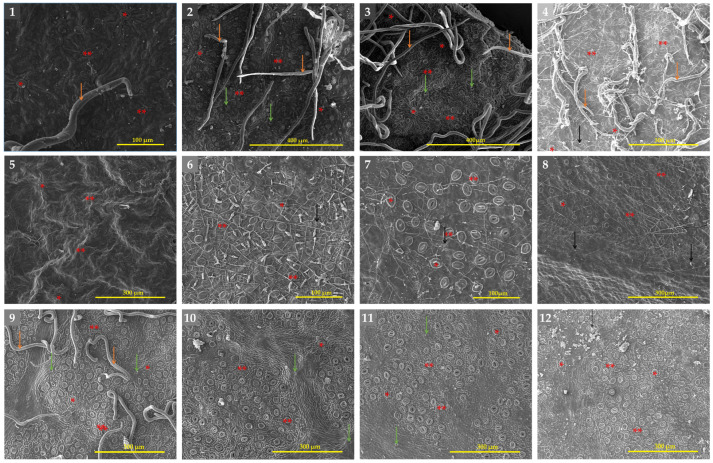
SEM micrographs of the adaxial surfaces of the leaves of *Eriobotrya* and *Rhaphiolepis* species: (**1**) *E. japonica*, (**2**) *E. × daduheensis*, (**3**) *E. tengyuehensis*, (**4**) *E. salwinensis*, (**5**) *E. crassifolia*, (**6**) *E. bengalensis*, (**7**) *E. henryi*, (**8**) *E. laoshanica*, (**9**) *R. umbellata*, (**10**) *R. integerrima*, (**11**) *R. × delacourii*, and (**12**) *R. jiulongjiangensis*. Designations: The orange arrows are non-glandular trichomes, the green arrows are bases of fallen trichomes, the black arrows are epicuticular waxes, a single asterisk indicate primary stomata, and double asterisks indicate secondary stomata. Scale bars: 100–400 µm.

Table 4 provides information on the quantitative and qualitative characteristics of the leaf epidermis, whereas Figure 7 shows selected species SEM images and Figures S2–S8 show all species within both genera. The abaxial epidermal cell shapes of *Eriobotrya* and *Rhaphiolepis* differed significantly; three categories were identified: irregular, with a parallel straight-curve (Group I), polygonal (Group II) within *Eriobotrya* species, and irregular, with a short straight-curve or irregular-undulate (Group III) within *Rhaphiolepis* species. The anticlinal walls of the pavement cells in most *Eriobotrya* species are not visible due to the cuticular microrelief that masks them (Figure 7), and the periclinal walls were either flat or convex. However, in *Rhaphiolepis*, only raised anticlinal walls, with convex periclinal walls were seen. The abaxial surfaces of the leaves were characterized by persistent non-granular multi-cellular trichomes, reported in seven species of *Eriobotrya* (*E. × daduheensis*, *E. malipoensis*, *E. japonica*, *E. prinoides*, *E. deflexa*, *E. salwinensis,* and *E. tengyuehensis*, mentioned in Figure S2: no. 4; Figure S4: nos. 9–10; Figure S5: no. 14; Figure S6: nos. 18–20, and two *Rhaphiolepis* species (*R. ferruginea*, and *R. umbellata*) (Figure S7: no. 26; Figure S7: no. 29)). In three species in *Eriobotrya* and three in *Rhaphiolepis*, the base of fallen trichome was not seen indicating these species are entirely glabrous throughout their life. Furthermore, if the trichome has fallen, it indicates that the species was initially tomentose, and later glabrescent when mature. Epicuticular wax deposits were identified in both genera, and the four forms of wax deposits seen were similar to those reported for the adaxial surfaces. Interestingly, five *Eriobotrya* species and three *Rhaphiolepis* species did not have epicuticular wax.

In terms of stomata size, *Eriobotrya* and *Rhaphiolepis* species displayed two main types: primary solitary stomata and secondary stomata (Table 4, Figure 7). Primary stomata are uncommon, typically large, and found alone in the center of a clump of medium-sized secondary stomata. The length and width of solitary stomata are much greater than those of secondary stomata. The average length and width of the primary stomata in *Eriobotrya* species varied from 22.95 to 35 × 15.54 to 26.64 µm, while those in *Rhaphiolepis* species ranged from 29.93 to 41.3 × 24.51 to 37.03 µm, respectively. *Eriobotrya* and *Rhaphiolepis* differed significantly in terms of secondary stomata size, secondary stomata outer ledge aperture size, and stomata ridge/rim width. *Eriobotrya* species had the smallest average secondary stomata size, outer ledge aperture size, and stomata rim width (17.09–26.84 × 13.39–20.93 µm, 13.12–22.79 × 10.06–16.04 µm, and 1.09–2.71 µm), while those in *Rhaphiolepis* species had the largest (29.3–35.07 × 23.87–30.94 µm, 22.84–29.11 × 16.21–23.64 µm, and 2.62–3.95 µm). In *Eriobotrya*, *E. elliptica* had the smallest secondary stomata (17.09 × 13.39 µm), while *E. prinoides* had the largest (26.84 µm). *E. hookeriana* had the widest width (20.93 µm), while *E. malipoensis* had the smallest (13.39 µm). In *Rhaphiolepis*, *R. ferruginea* had the smallest length of all the secondary stomata (29.3 µm) and the largest was in *R. × delacourii* (35.07 µm). *R. jiulongjiangensis* had the smallest width (23.87 µm), while *R. wuzhishanensis* had the largest (30.94 µm). *E. elliptica* had the smallest outer ledge aperture (13.12 × 10.06 µm), while *R. lanceolata* had the largest (29.11 µm). Furthermore, *E. malipoensis* had the shortest stomatal rim (13.39 µm), while *R. jiulongjiangensis* had the largest (3.95 µm). *Eriobotrya* species with a high stomata placement density per µm^2^, including *E. bengalensis*, *E. cavaleriei*, *E. deflexa*, *E. fragrans*, *E. malipoensis*, *E. japonica*, and *E. prinoides*, while *E. bengalensis* f. *angustifolia*, *E. crassifolia*, *E. henryi*, *E. tengyuehensis*, *E. serrata*, *R. indica*, *R. lanceolata*, *R. major*, *R. × delacourii*, *R. integerrima*, *R. wuzhishanensis*, and *R. jiulongjiangensis* had moderate placement density.

All *Eriobotrya* and *Rhaphiolepis* species studied have anomocytic stomata (as shown in Figure 7). *Eriobotrya* species had stomata on their abaxial surfaces, while *Rhaphiolepis* species lacked them. *Eriobotrya* stomata are evenly or regularly distributed in the areole regions, elliptical in shape, and located near or slightly above the pavement cells, whereas *Rhaphiolepis* stomata are unevenly or irregularly distributed, elliptical to round in shape, and located above the pavement cells. However, adaxial stomata are only recognized in *E. japonica* (Figure 7: no. 3). A regular arrangement of more robust radial folds was positioned adjacent to the prominent primary stomata. In *Eriobotrya* species (Figure 7: nos. 1–8), the nearest 2–3 parallel arrays of radial folds with all sides were discovered, whereas concentric to radially divergent folds were noted in *Rhaphiolepis* species, as depicted in Figure 7: nos. 9–12.

#### 3.2.3. Other Diagnostic Features

We reported other diagnostic micromorphological characteristics, including petiole surface sculpture, cross-sectional architecture, fruit surface, and the outer and inner surfaces of the fruit apical sepals, which can distinctly differentiate the two genera. The petioles of *Eriobotrya* species are glabrous or possess non-glandular trichomes (tomentose), exhibiting a smooth surface or long parallel ridges, with a circular outline in the cross-section, a well-developed circular arrangement of vascular bundles (amphicribral bundles), and possessing more mechanically supportive cells, including collenchyma and sclerenchyma (Figure S9). In contrast, the petioles of *Rhaphiolepis* species are either glabrous or hairy (non-glandular trichomes), characterized by an irregular polygonal shape with rounded ridges, triangular in the cross-section, and display a V-shaped or horizontally flattened arrangement of vascular bundles, with fewer supportive cells, comprising only collenchyma.

The fruit surface of *Eriobotrya* species is either glabrous or covered in non-glandular trichomes, featuring smooth to polygonal epidermal cells with parallel grooves, a smooth cuticle, and the presence of stomata (Figure S10). The outer surface sculpture of the apical fruit sepals of *Eriobotrya* species is consistently hairy (non-glandular trichomes), featuring irregular-undulate epidermal cells with cuticular striations, while epicuticular wax is absent. The inner surface is entirely glabrous, exhibiting irregular-undulate epidermal cells, depressed anticlinal walls, and convex periclinal walls; it also lacks epicuticular waxes, but containing stomata (Figure S10). In contrast, *Rhaphiolepis* species lack these characteristics. The principal diagnostic characteristics within and between genera are listed in the taxonomic treatment (for details, see Table 3, Table 4 and Table 5; Figure 4, Figure 5, Figure 6 and Figure 7). Conversely, the fruit surface of *Rhaphiolepis* is either glabrous or hairy (non-glandular trichome), characterized by irregularly polygonal epidermal cells with a rounded network of ridges, a glossy cuticle, and the presence of stomata (Figure S11).

**Taxonomic treatment**

Key to the genera *Eriobotrya* and *Rhaphiolepis* based on micromorphology.

1. Petioles exhibit a smooth surface or with long parallel ridges that are circular in the cross-section, and amphicribral bundles; the leaf adaxial surface is polygonal or irregular with long straight-curve cuticular folding patterns. The leaf abaxial surface is irregular, with long straight-curve or polygonal epidermal cells. Primary stomata are small, 22.95–35 × 15.54–26.64 µm; secondary stomata are small 17.09–26.84 × 13.39–20.93 µm. The outer stomata ledge aperture is small, 13.12–22.79 × 10.06–16.04 µm; the stomatal ridge rim is small, 1.09–2.71 µm. Fruit adaxial epidermal cells are smooth to polygonal with parallel grooves. The outer surface of the apical fruit sepals are non-granular trichomes, and the inner surface contains irregular-undulate epidermal cells. Laminar hydathodes, colleters and stomata are present near the leaf margin........................................................................ *Eriobotrya*
2. Petioles exhibit an irregularly polygonal surface with rounded ridges that are triangular in the cross-section, and V-shaped bundles; the leaf adaxial surface is irregular–polygonal, with smooth cuticular folding patterns, and the leaf abaxial surface is irregular, with short straight-curve or undulate epidermal cells. Primary stomata are large, 29.93–41.3 × 24.51–37.03 µm, and secondary stomata are large, 29.3–35.07 × 23.87–30.94 µm. The outer stomata ledge aperture is large, 22.84–29.11 × 16.21–23.64 µm; the stomatal ridge rim is large 2.62–3.95 µm. The outer surface of the fruit epidermal cells are irregularly polygonal with a rounded network of ridges; apical fruit sepals are absent. Laminar hydathodes, and stomata are absent near the leaf margin; colleters are usually absent, but were seen in one species on marginal teeth apices.......................................................................... *Rhaphiolepis*

**Generic description**

*Eriobotrya*

Leaf adaxial surface exhibited the following: Group 1: Polygonal (*E. crassifolia*, *E. × daduheensis*, *E. malipoensis*, *E. japonica*, *E. prinoides*, *E. cavaleriei*, *E. grandiflora*, *E. laoshanica*, *E. seguinii*, and *E. henryi*), Group 2: irregular epidermal cells (*E. bengalensis*, *E. condaoensis*, *E. bengalensis* f. *angustifolia*, *E. deflexa*, *E. elliptica*, *E. fragrans*, *E. petiolata*, *E. hookeriana*, *E. salwinensis*, *E. tengyuehensis*, *E. obovata* and *E. serrata*); depressed (for species with polygonal epidermal cells) or raised anticlinal walls (for species with irregularly shaped epidermal cells); smooth (for species with polygonal epidermal cells) or with long, straight-curve cuticular folding patterns (for species with irregularly shaped epidermal cells); fine microrelief striation flat patterns (for species with irregularly shaped epidermal cells) or with convex striation (for species with irregularly shaped epidermal cells); papillae were absent (*E. japonica*, *E. prinoides*, *E. laoshanica*, *E. salwinensis*, *E. tengyuehensis*, and *E. obovata*) or present to sparsely present (other species); the base of fallen trichomes were absent (*E. bengalensis*, *E. condaoensis*, and *E. grandiflora*) or present (other species); the epicuticular waxes were present in all species except *E. fragrans*; and well-developed laminar hydathodes were present in *E. fragrans*, *E. salwinensis*, *E. obovata*, and an unknown sample from Yunnan, China.

Leaf abaxial surfaces exhibited the following: anomocytic stomata; Group I: irregular, with long, straight-curve epidermal cells (*E. prinoides*, *E. tengyuehensis*, and *E. obovata*), Group II: polygonal epidermal cells (other species); raised anticlinal walls (*E. bengalensis*, *E. condaoensis*, *E. bengalensis* f. *angustifolia*, *E. deflexa*, *E. × daduheensis*, *E. prinoides*, *E. cavaleriei*, *E. salwinensis*, *E. tengyuehensis*, and *E. obovata*) or depressed anticlinal walls (other species); flat periclinal walls (*E. crassifolia*, *E. grandiflora*, *E. elliptica*, *E. serrata*, *E. laoshanica*, *E. seguinii*, and *E. henryi*) or convex periclinal walls (other species); the base of fallen trichomes were absent (*E. bengalensis*, *E. condaoensis*, *E. cavaleriei*, and *E. elliptica*) or present (other species); non-glandular trichomes were present in *E. × daduheensis*, *E. deflexa*, *E. japonica*, *E. prinoides*, *E. malipoensis*, *E. salwinensis*, and *E. tengyuehensis*; epicuticular waxes were absent in *E. crassifolia*, *E. condaoensis*, *E. deflexa*, *E. petiolata*, and *E. prinoides*, or present (other species).

Stomatal distribution was as follows: amphistomatic, hypostomatic, and evenly/regularly distributed.

Stomata size and frequency: Primary solitary stomata were 22.95–35 × 15.54–26.64 µm. Secondary stomata were 17.09–26.84 × 13.39–20.93 µm; outer stomatal ledge apertures were 13.12–22.79 × 10.06–16.04 µm. The widths of stomatal ridge rims were 1.09–2.71 µm, and 134–578/0.09 µm^2^.

Petioles outlines included the following: glabrous or non-glandular trichomes (tomentose), surface sculptures were smooth or had long parallel ridges that were circular in the cross-section, with a well-developed circular arrangement of vascular bundles (amphicribral bundles), and more mechanistically supportive cells including collenchyma and sclerenchyma.

Fruit surfaces were as follows: glabrous or hairy (non-glandular trichomes), containing smooth to polygonal epidermal cells, with parallel grooves; and smooth cuticles, with the presence of stomata.

The outer surfaces of the apical fruit sepals were as follows: hairy (non-glandular trichomes), irregular-undulate epidermal cells with cuticle striation, and epicuticular wax was absent.

The inner surfaces of the apical fruit sepals were glabrous overall, with irregular-undulate epidermal cells, depressed anticlinal walls, and convex periclinal walls; epicuticular waxes were absent and stomata were present.

Colleters and stomata at the leaf margin presented as follows: colleters were along the leaf marginal teeth apices, with stomata aggregated around the margin and in the sinuses between the leaf teeth in the majority of *Eriobotrya* species.

*Rhaphiolepis*

Leaf adaxial surfaces exhibited the following: Group 3: overall irregular-polygonal or thin-verrucous epidermal cells; depressed anticlinal walls except in *R. major* and *R. wuzhishanensis* (raised); smooth cuticular folding patterns except in *R. major* and *R. wuzhishanensis,* which had an irregular surface, with short, straight-curve cuticular folding; fine flat microrelief (*R. indica*, *R. lanceolata*, *R. umbellata*, *R. umbellata* var. *liukiuensis*, *R. × delacourii*, and *R. integerrima*) or with convex striation patterns (*R. major*, *R. wuzhishanensis*, *R. jiulongjiangensis*, and *R. ferruginea*); papillae were absent (*R. indica*, *R. lanceolata*, *R. jiulongjiangensis*, *R. ferruginea*, and *R. × delacourii*) or present to sparsely present (other species); the base of fallen trichomes was present (all species); epicuticular waxes were present (all species); and laminar hydathodes were not seen in the studied species.

Leaf abaxial surfaces exhibited the following: anomocytic stomata; Group III: irregular, with short straight-curve or undulate epidermal cells (all species) except *R. major* (polygonal-undulate or irregular undulate); raised anticlinal walls (all species); convex periclinal walls (all species); the base of fallen trichomes was absent (*R. jiulongjiangensis*) or present (other species); non-glandular trichomes were present in *R.umbellata*, *R. wuzhishanensis*, and *R. ferruginea*; epicuticular waxes were absent in *R.umbellata*, and *R. major* or present (other species).

Stomatal distribution was as follows: hypostomatic, and unevenly/irregularly distributed.

Stomata size and frequency: Primary solitary stomata were 29.93–41.3 × 24.51–37.03 µm. Secondary stomata were 29.3–35.07 × 23.87–30.94 µm; outer stomata ledge apertures were 22.84–29.11 × 16.21–23.64 µm. The widths of stomatal ridge rims were 2.62–3.95 µm, and 334–467/0.09 µm^2^.

Petioles outlines were as follows: glabrous or hairy (non-glandular trichomes), with irregularly polygonal surface sculptures with rounded ridges that are triangular in the cross-section, a V-shaped or horizontally flattened arrangement of vascular bundles, and less supportive cells with collenchyma only.

Fruit surfaces were as follows: glabrous or hairy, epidermal cells were irregularly polygonal with a rounded network of ridges, glossy cuticles, and the presence of stomata.

Outer surfaces of the apical fruit sepals: lack all species.

Inner surfaces of the apical fruit sepals: lack all species.

Colleters and stomata at the leaf margin and apex presented as follows: a colleter was located along the leaf margin apex in *R. lanceolata*, and in the sinuses between the leaf teeth in *R. jiulongjiangensis*, without adjacent stomata, while another colleter was found at the leaf apex in *R. indica*, *R. lanceolata*, and *R. salicifolia*.

#### 3.2.4. Principal Component Analysis (PCA) and Cluster Analysis (CA)

**Figure 8:**
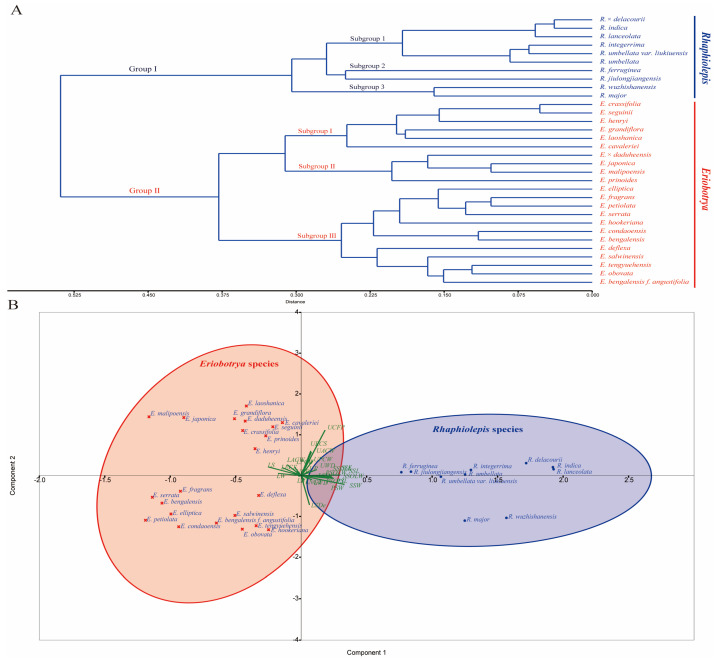
(**A**) Cladogram of 32 *Eriobotrya* and *Rhaphiolepis* species based on 26 micromorphological variables. (**B**) Principal Component Analysis graph showing the contributions of these variables to the explanation of leaf micromorphological variation. The Red cross indicate *Eriobotrya* species, and Blue circle indicate *Rhaphiolepis* species.

The principal component analysis plot revealed a clear subdivision of the studied species of *Eriobotrya* and *Rhaphiolepis* (Table S4) into two distinct groups, which was predominantly driven by principal components 1 and 2 (PC1 and PC2) (Table S6). Furthermore, the first two components from the PCA accounted for 53.292% of the total variation (Table S6). The first principal component accounted for 39.86% of the variation, with epidermal cell shapes, anticlinal and periclinal walls, cuticular folding, stomatal complexes and distribution as the most important variables, while the second component accounted for 13.432% of the variation, with epidermal cell shapes, anticlinal and periclinal walls on both surfaces, and non-glandular trichomes and stomatal complexes identified as the most important variables (Figure 8). The result based on nine stomatal variables also showed a clear subdivision of the studied species of *Eriobotrya* and *Rhaphiolepis* into two distinct groups (Figure S12).

Species from the two genera were divided into two main groups based on the cluster analysis of the 26 leaf morphoanatomy variables (Table 1, Table 3, Table 4, Table 5 and Table S4, Figure 8), while the analysis based on stomatal complexes is presented in Table 5, Tables S7 and S8, and Figure S12. In the cluster analysis, Group I included all 10 of the *Rhaphiolepis* species, and was further separated into two subgroups 1, 2, and 3: subgroup 1 comprised *R. × delacourii*, *R. indica*, *R. lanceolata*, *R. integerrima*, *R. umbellata*, and *R. umbellata* var. *liukiuensis*; subgroup 2 comprised *R. ferruginea*, and *R. jiulongjiangensis*; whereas subgroup 3 included *R. major*, and *R. wuzhishanensis*. Group II included 22 species of *Eriobotrya*, and was further divided into three subgroups, I, II, and III: subgroup I had six species, comprising *E. crassifolia*, *E. laoshanica*, *E. cavaleriei*, *E. grandiflora*, *E. seguinii*, and *E. henryi*. Subgroup II had 5 species, including *E. × daduheensis*, *E. japonica*, *E. malipoensis*, and *E. prinoides*, while subgroup III had 12 species, including *E. bengalensis*, *E. bengalensis* f. *angustifolia*, *E. condaoensis*, *E. elliptica*, *E. serrata*, *E. salwinensis*, *E. fragrans*, *E. deflexa*, *E. tengyuehensis*, *E. obovata*, *E. petiolata,* and *E. hookeriana*.

## 4. Discussion

Numerous documented instances among closely related extant genera suggested that the tribe (Maleae) of Rosaceae experienced extensive intergeneric and trigeneric hybridization, ancient and recent radiations, incomplete lineage sorting (ILS), polyploidy events, the loss of gene copies following duplication events, or ancient divergence among certain clades [7,26,28,37,44,74,75,76,77]. It is possible that Maloideae evolved polychotomously from an allopolyploid gene pool that resulted from hybridization between an Amygdaloideae ancestor (x = 8) and a Spiraeoideae ancestor (x = 9) [8,78,79] or that the original rootstock was solely spiraeoid [80]. The ancestor of Maleae (x = 17) and Gillenieae (x = 9) may have originated through hybridization between two distantly related Amygdaloideae lineages, according to a recent phylogenomic study [81]. In addition, it has been proposed that the genera *Phippsiomeles* B.B.Liu & J.Wen [82], *Micromeles* Decne., *Pseudocydonia* (C.K.Schneid.) C.K.Schneid. [28], and *Sorbus* L., and *Micromeles* [83] have hybrid origins. *Eriobotrya* has long been thought to be prone to hybridization [84,85], and despite the existence of intergeneric hybrids [28,38,84], almost all studies involving the genera *Eriobotrya* and *Rhaphiolepis* have found close genetic and morphological relationships [26,28,30,31,33,34,36]; however, there is no reason to combine them [24,44]. There may be incongruence between these disparate data sources because sequences from different genomes exhibit different inheritance patterns (e.g., nuclear genes are for biparentally inherited, plastid genes and mitochondrial genes are for maternally inherited genes) [86,87,88,89]. At a deeper level, in *Eriobotrya*, Liu et al. [40] documented a hybridization event involving *Rhaphiolepis* using a phylogenomic approach. Furthermore, Chen et al. [46] found numerous occurrences of multiple ancient hybridization events, and chloroplast capture events within *Eriobotrya*. When the evolutionary histories of various genes conflict, hybridization is frequently identified by gene tree discordance [90]. These discrepancies could be explained by gene flow (introgression, allopolypoloidy, hybridization), mitochondrial or chloroplast capture events, horizontal gene transfer, ILS, and errors in gene tree estimation.

### 4.1. Phylogenetic Relationships

The phylogenetic status of the two genera within the Maleae tribe has been contentious, and their evolutionary relationships remain similarly contentious. Phylogenetic analyses utilizing nuclear genome data have affirmed the monophyly of both genera [25,26,29,34,37], whereas alternative studies employing nrDNA and cpDNA data have classified them as paraphyletic [40,44,55]. Integrative taxonomy, which synthesizes information from several fields including morphology, phylogenomics, cytology, and ecology, has been advocated for as a normative approach in taxonomic research [91]. This study employs a multi-evidence approach, utilizing molecular (nrDNA, and mtDNA) and micromorphological analyses (26 variables) for the first time to clarify the phylogenetic relationships between two genera, elucidate the micromorphological patterns of variation, and identify the most informative features for differentiating the two genera and assessing their taxonomic relationship. The phylogenetic trees (Figure 2 and Figure 8) derived from complete nrDNA sequences and micromorphological variables support the monophyly of each genus, with high support for *Rhaphiolepis* (BS = 100%, PP = 1.0) and moderate to low support for *Eriobotrya* (BS = 51%, PP = 0.67). These findings align with previously published molecular research [33,34,35,36,37,92] and morphological studies [7,9,11,12,15,17,19,20,23,38,47,52], although the relationships among and within the clades varied in each analysis. Two principal clusters within *Eriobotrya* (25 species) and two within *Rhaphiolepis* (14 species) were discerned. The phylogenomic topologies established herein align with previously documented phylogenetic relationships [33,34], which delineated the relationships among 17 and 18 *Eriobotrya* species, respectively. The majority of interspecies relationships within *Eriobotrya* (22 species) have been elucidated and validated through recent nrDNA (ITS) research [36], which will not be reiterated here; instead, this discussion will concentrate on problematic species, using both molecular and morphological evidence. Previously, a robust interspecies or possibly infraspecies relationship was documented between *E. fragrans* and *E. cavaleriei* [27,33,34] and it is addressed herein. Our analysis of nrDNA indicated that *E. cavaleriei* from GenBank and *E. fragrans* formed a sister clade to *E. deflexa*, while another sample of *E. cavaleriei* from Chongqing formed a clade with *E. elliptica*. Morphologically, *E. fragrans* closely resembles *E. deflexa* but differs from it by the presence of non-glandular trichomes on the abaxial surface, epicuticular wax deposits on both surfaces, and stomatal complexes (Table 3, Table 4 and Table 5; Figures S2 and S3). *E. fragrans* is further distinguished from *E. cavaleriei* by several traits (Table 3, Table 4 and Table 5; Figure 8, Figures S3 and S5), including irregular epidermal cell shapes, with cuticle striation patterns (vs. a polygonal shape, without cuticle striation in *E. cavaleriei*), raised anticlinal and convex periclinal walls (vs. depressed and flat walls in *E. cavaleriei*), as well as variations in the epicuticular waxes, stomatal complexes, and frequencies (Table 3, Table 4 and Table 5). *E. × daduheensis*, *E. prinoides*, *E. japonica*, *E. malipoensis*, and *E. tengyuehensis*, constituted a cluster in nrDNA, mtDNA, and micromorphological analyses gene trees, signifying close relationships, consistent with recent publications [33,34,36,55,92]. All of these species exhibited polygonal epidermal cell morphology, depressed anticlinal cell walls, smooth periclinal walls, and non-glandular simple trichomes (indumentum) on both surfaces. In contrast, *E. prinoides* and *E. tengyuehensis* displayed an irregular abaxial surface characterized by long, straight-curve epidermal cells, raised anticlinal walls, and convex periclinal walls. Dong et al. [34] showed that *E. prinoides* is closely related to *E. serrata* and *E. elliptica*. Our findings however, revealed a discrepancy; morphological and molecular evidence corroborated the aforementioned relationships, as cited above, while our results aligned with Dong et al. regarding the phylogenetic positioning of *E. bengalensis*, *E. salwinensis*, *E. bengalensis* f. *angustifolia*, and *E. obovata,* which, with the addition of *E. crassifolia, E. elliptica, E. petiolata and E. cavaleriei,* form a closely related group consistent with other studies [33,36].

In the genus *Rhaphiolepis*, the designation *R. jiulongjiangensis* was omitted from the Flora of China [15], which indicated the necessity for further investigation. However, with the increasing number of species, we have confirmed its phylogenetic position, establishing a clade with *R. ferruginea* var. *serrata* in both nrDNA and mtDNA (Figure 2 and Figure 3). Micromorphologically (Figure 8), *R. jiulongjiangensis* is closely related to *R. ferruginea*, forming a clade but differing due to the absence of fallen trichomes (glabrous) on the adaxial surface (vs. their presence in *R. ferruginea*), and primary stomatal dimensions of 32.04 × 24.51 (vs. 29.93 × 28.29 in *R. ferruginea*) (Table 3, Table 4 and Table 5; Figure S7). Moreover, recent research indicated that *R. jiulongjiangensis* constituted a distinct clade, and established a sister group with other *Rhaphiolepis* species [36], which could aid in understanding the evolution of *Rhaphiolepis* and its relationship with *Eriobotrya*. Our findings supported its evolutionary position, and morphological affinities with the closest species.

*Rhaphiolepis × delacourii* constituted a clade with *R. indica* var. *shilanensis* (Figure 2), while morphologically it is closely related to *R. indica* (Figure 8) but differs in having polygonal-undulate epidermal cells (vs. irregular, small, straight-curve cells in *R. indica*), and distinct stomatal complexes (Table 3, Table 4 and Table 5). Huang & Li [93] regarded *R. umbellata* var. *liukiuensis* as a synonym of *R. umbellata*, indicating that they differ in their leaf margins. Our findings showed that *R. umbellata* var. *liukiuensis* formed a sister clade with other *Rhaphilepis* species, including *R. major*, in the nrDNA analysis, whereas micromorphological and mtDNA phylogenies (Figure 3 and Figure 8) indicated its sister clades *R. integerrima*, and *R. umbellata*. Nonetheless, it is distinguishable from *R. umbellata* by the presence of distinct trichomes and epicuticular waxes on both surfaces. We additionally elucidated the relationships among 17 species of *Eriobotrya* and *Rhaphiolepis*, specifically *E. laoshanica*, *E. crassifolia*, *E. sp1* to *E. sp5*, *E. hookeriana*, *R. indica* var. *shilanensis*, *R. jiulongjiangensis*, *R. umbellata* var. *liukiuensis*, *R. × delacourii*, and *R. wuzhishanensis*.

### 4.2. Conflict

Although *Eriobotrya* monophyly was well supported in both nuclear and leaf micromorphology phylogenies (Figure 2 and Figure 8), we discovered mito-nuclear discordance within this genus (Figure 3). Conflicts between organelle genomic data and nrDNA are common in the *Eriobotrya*-*Rhaphiolepis* clade [40,46], and have been reported in many Rosaceae genera, including *Prunus* L. [94], *Cotoneaster* Medik. [95], and *Potentilla* L. [96]. The cyto-nuclear discordance phenomenon is difficult to avoid due to the inherent difference of evolutionary histories between nuclear and cytoplasmic genomes. We found topological congruences between nrDNA and micromorphology, as well as major inconsistencies in the partial mtDNA. Although the mtDNA gene tree derived from 52 protein-coding genes shows generally low resolution (with weakly supported nodes) due to low sequence variability, the discordant clade comprising the four *Eriobotrya* species nested within *Rhaphiolepis* is significant, with support ranging from low (for *E. hookeriana*: BS = 52%, PP = 0.93) to strong confidence (for *E. fragrans*, *E. deflexa* and *E. laoshanica*: BS = 94%, PP = 1.0) (Figure 3). Tree topological incongruence can be caused by a variety of biological patterns, including gene flow (e.g., hybridization and introgression), mitochondrial capture events, gene choice, or technological causes (e.g., insufficient data) [86]. In both nrDNA and micromorphological investigations, these species formed a distinct clade within *Eriobotrya*. First, *E. deflexa* has close relationships with *E. fragrans* in both nrDNA and mtDNA trees (with BS–PP 97 and 1) (Figure 2 and Figure 3), which is consistent with recently published phylogenetic studies [34,36,46], though these species morphologically differ from *Rhaphilolepis* by having abaxial epidermal cell shapes, primary and secondary stomata, outer primary and secondary ledge apertures, stomata distribution, fruit surfaces, and the outer and inner surfaces of the fruit apical sepals (Table 3 and Table 5; Figures S2–S8). Second, *E. laoshanica* formed a sister clade with *E. deflexa* and *E. fragrans*; although the morphological tree revealed that it formed a clade with *E. grandiflora*, they differed from each other in terms of stomatal complexes and frequency. Furthermore, this species is distinguished from *Rhaphiolepis* species by its abaxial epidermal cell shapes, stomatal complexes, fruit surface, and the sculpture of the outer and inner surfaces of the fruit apical sepals. Third, *E. hookeriana* formed a sister clade with *E. henryi* and *E. seguinii* in Clade I of the nrDNA gene tree, and the morphological tree revealed that it formed a sister clade with *E. elliptica*. Additionally, *E. hookeriana* species differ from *Rhaphiolepis* by having an abaxial leaf surface, fruit surfaces, and the outer and inner surfaces of the fruit apical sepals (see Table 3, Table 4 and Table 5; Figures S2–S8). The majority of the species relationships within the genera *Eriobotrya* and *Rhaphiophis* are consistent with those of the nrDNA results. For example, *E. prinoides*, *E. japonica*, *E. malipoensis*, and *E. tengyuehensis* formed a clade, and *E. elliptica*, and *E. petiolata* formed a clade, aligning with prior research. Furthermore, the resulting network revealed that these species have a complex evolutionary history (Figure S1); their presence increases network complexity, which could be due to frequent hybridization from *Rhaphiolepis* into *Eriobotrya* or a lack of informative characters. The removal of these species resulted in no reticulation, confirming their involvement in the evolutionary debate. Previous studies [40] found cyto-nuclear conflicts in both genera, indicating hybridization. Compared to hybridization or introgression, fewer studies have reported cyto-nuclear discordance attributed primarily to ILS. This is most likely because ILS is more difficult to identify and less appealing than the rush of cytoplasmic genome capture or nuclear gene flow among phylogenetically distant species, which may produce more eye-catching topologically incongruent patterns [87]. Furthermore, phylogenetic trees built on diverse data matrices frequently produce inconsistent results [88]. Such different topologies can perplex taxonomists, perhaps leading to inaccurate taxonomic treatments.

### 4.3. Morphological Relationships

The micromorphological structure of leaf blades displays variability among different genera, while remaining rather uniform within a species, thereby facilitating the use of these traits for taxonomic classification [97]. Comprehensive investigations of leaf surface micromorphology in *Aronia* Medik., and *Pourthiaea* Decne., by Vinogradova [98], distinguished the genera by the presence of colleters on the midrib of adaxial surfaces and cuticular folding. Song and Hong [68], and Song et al. [69] distinguished the tribes Spiraeeae, Sorbarieae, and Neillieae by stomatal complexes, surface anticlinal walls, wax deposits, and trichome diversity; the genus *Cotoneaster* was distinguished by Niaki et al. [99] by epidermal cells, anticlinal walls, stomatal types, epicuticular waxes, cuticle density, and trichomes. Dryadoideae (Rosaceae) was distinguished by Babosha et al. [97] by stomata, cuticular folding patterns, and trichomes with hydathodes. Genera in the Rosaceae can be distinguished by cuticular folding patterns and microstructure [70]. Nonetheless, there is limited evidence concerning the micromorphology of the leaf epidermises, petioles, pollens, fruits, and fruit apical sepals of *Eriobotrya* and *Rhaphiolepis*, which is crucial for classification. Additionally, anomocytic stomata were identified in the Rosaceae [50], with the first documentation in *E. japonica* by de Sauza et al. [54], which is consistent with the present study. The anatomy and significance of hydathodes in the Rosaceae remain largely unexamined. Guttation has been documented in Rosoideae and Spiraeoideae, while hydathodes, or water pores, and colleters were documented exclusively in six genera (*Eriobotrya*, *Crataegus* L., *Malus* Mill., *Mespilus* L., *Amelanchier* Medik., and *Sorbus* L.) in the tribe Maleae [70,100]. Lippmann [101] observed the presence of water pores but the absence of epithem in *Malus floribunda*, concluding that neither structure is found in *Crataegus*, *Pyrus*, *Cotoneaster*, and *Malus spectabilis*. The present investigation revealed that the adaxial surface of *Eriobotrya* had well-developed laminar hydathodes (water pores and epithem), in accordance with Lersten and Curtis [102] (Figure 5), as well as colleters at the apex of leaf marginal teeth and in the sinuses between the leaf marginal teeth (Figure 6), and surrounding stomata were observed on the leaf teeth. In *Rhaphiolepis* species, colleters were found at the leaf margin apex in *R. lanceolata*, while in *R. jiulongjiangensis*, they were noted in the sinuses between the leaf teeth, without surrounding stomata on the adaxial surface, near the margin apex, or at the leaf apex. It is important to highlight that the stomata are present at the leaf margin apex in *Eriobotrya* species while absent in *Rhaphiolepis*; while the same structures were previously identified as water pores in various genera of Rosaceae [102,103], we refer to them here as adaxial stomata. Colleters of the same type were documented on the leaf serrations of *Prunus* [104] and *Eriobotrya japonica* [105]. Colleters are secretory structures that assist in safeguarding meristems and growing organs by preventing dehydration [106]. Moreover, large, flat marginal glands and dark punctate spots on leaves were observed in both genera (Figure S13), comprising a glandular zone and its associated parenchyma cell stalk tissues [107]. These structures were exclusively identified in *Prunus* [108], which secretes sugars and attracts ant visitation during active secretion [109]. Comprehensive descriptions of hydathodes, colleters, or glandular anatomy employing modern approaches are necessary to determine the functionality of the reported structures for guttation and secretions. Thus, we determined that the adaxial epidermal cells, anticlinal walls, periclinal walls, abaxial epidermal cells, cuticular folding, stomatal complexes and distribution, colleters, hydathodes, stomata near the leaf margin apex, petiole surface sculpture and cross-section outline, fruit surface sculpture, and the outer and inner surfaces of the fruit apical sepals are critical diagnostic traits for the classification of these two genera in the Rosaceae. Additionally, within these genera, the shape of the epidermal cells, anticlinal walls, non-glandular trichomes, and stomatal complexes are notable features. It has also been established that a number of micromorphological characteristics, such as quantitative and qualitative variables, were helpful in differentiating between and within the two genera. This study is the first to demonstrate that using SEM in conjunction with DNA evidence for taxonomic purposes reflects the benefits of detailed morphological data and large-scale analysis. These data can also be a valuable source of knowledge and a thorough archive of micromorphological databases with a wide range of uses in teaching and research.

### 4.4. Morphological Comparison with Previous Studies

Prior research showed that *E. henryi*, *E. seguinii*, *E. bengalensis* f. *angustifolia*, *E. salwinensis*, and *E. obovata* formed a sister clade to the *Rhaphiolepis* clade, with robust support values [40]. Moreover, the author determined that the apical sepals of the fruit in *E. henryi* are not persistent, and the lateral veins of the leaves in both *E. henryi* and *E. seguinii* exhibit curvatures. This leads to the classification of both genera under a single genus based on two synapomorphies, as outlined by Aldarsoro et al. [24]: a lack of endosperm and the existence of rounded or broad elliptic seeds. Our finding based on nrDNA showed that *E. seguinii*, *E. henryi*, and *E. hookeriana* formed a closely related group, considered to be the earliest diverging extant lineage within *Eriobotrya.* This agrees with earlier studies based on molecular evidence [27,33,35,55]. Furthermore, we verified here (see Figure S10: nos. 9–12; Figure S13: nos. 8–16) that a persistent sepal effectively distinguished the two genera, including *E. seguinii*, and *E. henryi*, along with the fruit adaxial surface sculpture and petiole cross-section (Figures S9 and S10). Based on leaf venation, taxonomic studies [7,8,9,15] indicated that *Eriobotrya* displays both camptodromous and craspedodromous leaf venation patterns. Furthermore, Aldarsoro et al. [24] previously determined that the absence of endosperm along with rounded or broadly elliptic seeds in one *Eriobotrya* and one *Rhaphiolepis* species, which constituted a sister clade in the phenogram, differentiated them from other Maloideae genera characterized by 8 to 18 seed coat layers and broadly elliptic or oval seeds [6,7]. It is noteworthy that both genera possess proportionally larger seeds compared to other Maloideae and reside in the understory of tropical or subtropical evergreen forests with diminished light intensity [22,110]. Furthermore, *Eriobotrya* cotyledons exhibit photosynthesis, turning green after germination [111]. Gu and Spongberg [9,15] reported obovoid, ovoid to ovoid-globose, elliptic, globose, or pyriform to subglobose fruit shapes within *Eriobotrya* and exclusively globose fruit shapes in *Rhaphiolepis* (Figure S13). Robertson et al. [7] recognized both genera as distinct, noting that “the narrow petals and elongated panicles resemble those of *Amelanchier* while the thin endocarp and one or two seeds are similar to *Eriobotrya*, except that the calyx is quickly deciduous as a unit, leaving an annular ring” (see Figure S13: nos. 8–16). They further observed that sclereids are absent to densely and evenly distributed in *Eriobotrya*, in contrast to the numerous, scattered sclereids in *Rhaphiolepis*, which aligns with the previous findings [6,8].

Overall, congruences in morphological and molecular data were observed in the studied genera, reinforcing traditional taxonomy. The morphological analysis revealed that none of the examined characteristics of the four distinct *Eriobotrya* species (including abaxial and adaxial leaves, stomatal complexes, petiole cross-sectional outlines, fruit surface, and the outer and inner surfaces of the apical fruit sepals) were similar to those of *Rhaphiolepis*. The observed mtDNA incongruence is most consistent with mito-nuclear discordance potentially resulting from ancient hybridization, mitochondrial capture events, incomplete lineage sorting, gene choice, or technological causes (e.g., insufficient data). Furthermore, the slow evolutionary rate and lack of relevant features in the mtDNA protein coding genes employed in this study preclude a robust phylogenetic reconstruction, making this genome particularly sensitive to representing ancient hybridization events. Future studies incorporating whole genome sequencing may offer additional insight into the intricate evolutionary history of this significant group, even though the creation of new sequence data will continue to be crucial.

## 5. Conclusions

The classification of *Eriobotrya* and *Rhaphiolepis* as distinct, monophyletic genera is supported by congruent evidence from the complete nuclear ribosomal DNA sequences (nrDNA) and a comprehensive analysis of micromorphological features (including the diagnostic features of leaves, petioles, fruits, apical sepals of the fruit, etc.). We recommend that *Eriobotrya* and *Rhaphiolepis* be classified as distinct genera. Leaf epidermal micromorphological features, such as epidermal cell shapes and the anticlinal walls on both surfaces, cuticular folding, stomatal complexes and distribution, colleters, hydathodes, amphistomatic leaves, stomata located near the leaf margin, petiole surface sculpture and cross-section outline, fruit surface sculpture, and the outer and inner surfaces of the fruit apical sepals, were effective for distinguishing and classifying both *Eriobotrya* and *Rhaphiolepis* and are consistent with the well-supported phylogeny from nrDNA. Furthermore, epidermal cell shapes, anticlinal walls, non-glandular trichomes, and stomatal complexes were important characteristics for distinguishing species within these genera. The outcomes of this study support the idea that SEM imaging of leaf, petiole, and fruit micromorphology is advantageous for delineation at the generic level, and upholds them as distinct genera. Furthermore, this approach enabled us to collect a vast quantity of quantitative and qualitative data on the leaves, petioles, and fruit surfaces with high accuracy, which promises to be very useful in resolving the taxonomic classification of *Eriobotrya* and *Rhaphiolepis*. The aberrant mtDNA signal, which showed four distinct *Eriobotrya* species, formed a sister group within the *Rhaphiolepis* clade, inferred a complex evolutionary history involving ancient hybridization, mitochondrial capture, or phylogenetic error. While molecular (nrDNA) and micromorphological evidence of four distinct *Eriobotrya* species clearly supports their classification as *Eriobotrya*, future studies that include whole genome sequencing may shed further light on the complex evolutionary history of this key clade.

## Figures and Tables

**Table 1:** Micromorphological features of *Eriobotrya* and *Rhaphiolepis* species under scanning electron microscopy.

No.	Characters	Code
1	Leaf length, LL (cm)	<8 (0); 9–18 (1); 19–28 (2); >28 (3)
2	Leaf width, LW (cm)	<5 (0); 5–10 (1); >10 (2)
	Adaxial surface (U)	
3	Epidermal cell shape (UECS)	Irregular to straight-curve (0); Polygonal (1)
4	Anticlinal wall (UACW)	Raised (0); Depressed (1)
5	Cuticular folding pattern (UCFP)	Long, straight-curve (0); Small, straight-curve (1); Smooth (2)
6	Periclinal wall (UPCW)	Convex (0); Flat (1)
7	Papillae (UP)	Present (0); Sparsely Present (1); Absent (2)
8	Base of fallen trichome (UBT)	Present (0); Absent (1)
9	Epicuticular wax deposits (UWDs)	Granule (0), sparsely granule (1); Rodlets (2); Sparsely rodlets (3)
	Abaxial surface (L)	
10	Epidermal cell shape (LECS)	Irregular, with long straight-curve (0); Irregular, with small straight/undulate curve (1); Polygonal (2)
11	Anticlinal wall (LACW)	Raised (0); Depressed (1)
12	Periclinal wall (LPCW)	Convex (0); Flat (1)
13	Base of fallen trichome (LBT)	Present (0); Absent (1)
14	Persistent non-glandular trichome (LNGT)	Present (0); Absent (1)
15	Epicuticular wax deposits (LWDs)	Granule (0), Sparsely granule (1); Rodlets (2); Sparsely rodlets (3)
	Primary Stomata	
16	Stomata distribution (LSDi)	Evenly (0); Unevenly (1)
17	Length of stomata (PSL) (µm)	<24–30 (0); >30–36 (1); >36 (2)
18	Width of stomata (PSW) (µm)	<15–22 (0); >22–29 (1); >29 (2)
19	Length of outer ledge aperture (PSOLL) (µm)	<20–26 (0); >26–31 (1); >31 (2)
20	Width of outer ledge aperture (PSOLW) (µm)	<9–15 (0); >15–22 (1); >22 (2)
	Secondary Stomata	
21	Length of stomata (SSL) (µm)	<17–23 (0); >23–29 (1); >29 (2)
22	Width of stomata (SSW) (µm)	<13–19 (0); >19–25 (1); >25 (2)
23	Width of ridge rim (SSE) (µm)	<1–1.5 (0); >1.5–2.5 (1); >2.5 (2)
24	Length of outer ledge aperture (PSOLL) (µm)	<17–22 (0); >23–28 (1); >28 (2)
25	Width of outer ledge aperture (SSOLW) (µm)	<10–15 (0); >15–20 (1); >20 (2)
26	Number of stomata per µm^2^ (LSDe)	<150–300 (0); >300–450 (1); >450 (2)

**Table 2:** Sequence information and variability of nuclear vs. mitochondrial sequences in *Eriobotrya* and *Rhaphiolepis* species.

Sequence Information	Nuclear Sequences (nrDNA)	Mitochondrial Sequences (mtDNA)
Total number of samples (n)	59	43
Number of species (n)	39 (E: 25 & R: 14)	38 (E: 25 & R: 13)
Alignment length (bp)	39,394	42,440
Constant sites (bp)	37,363	41,848
Variable sites (bp)	2031	592
Parsimony-information sites (bp)	1079	349
% pairwise identity	71.8	93.5
% identical sites	13.4	66.8
GC contents (%)	55	43.5

n = number, bp = base pairs, % percentage, E = *Eriobotrya*, R = *Rhaphiolepis*.

**Table 3:** Adaxial quantitative and qualitative variables assessed for study on the micromorphological features of *Eriobotrya* and *Rhaphiolepis* species under scanning electron microscopy.

No.	Taxon	LL (cm)	LW (cm)	UECSs	UACWs	UCFPs	UPCWs	UP	UBTs	UWDs
1	*E. bengalensis*	7–20	2.5–6	irregular	raised	long, straight-curve	ridge-convex striated	present	absent	granules
2	*E. bengalensis* f. *angustifolia*	7–15	2–8	irregular	raised	long, straight-curve	ridge-convex striated	present	present	sparse rodlets
3	*E. condaoensis*	8–12	4–6	irregular	raised	long, straight-curve	ridge-convex striated	sparsely present	absent	sparse granules
4	*E. deflexa*	10–20	3–7	irregular	raised	long, straight-curve	ridge-convex striated	sparsely present	present	sparse granules
5	*E. elliptica*	16–25	4–9	irregular	raised	long, straight-curve	ridge-convex striated	present	present	rodlets
6	*E. fragrans*	7–15	2.5–5.5	irregular	raised	long, straight-curve	ridge-convex striated	sparsely present	present	absent
7	*E. petiolata*	16–22	6–9	irregular	raised	long, straight-curve	ridge-convex striated	present	present	granules
8	*E. hookeriana*	12–25	4–8	irregular	raised	long, straight-curve	ridge-convex striated	present	present	granules
9	*E. salwinensis*	10–20	2.5–6.5	irregular	raised	long, straight-curve	ridge-convex striated	absent	present	sparse rodlets
10	*E. tengyuehensis*	10–18	4–7	irregular	raised	long, straight-curve	ridge-convex striated	absent	present	granules
11	*E. obovata*	7–16	2–6	irregular	raised	long, straight-curve	ridge-convex striated	absent	present	rodlets
12	*E. serrata*	10–25	4–13	irregular	raised	long, straight-curve	ridge-convex striated	sparsely present	present	sparse granules
13	*E. crassifolia*	9–12	2.5–3.5	polygonal	depressed	smooth	flat to thin verruca	sparsely present	present	sparse granules
14	*E.* *× daduheensis*	14–21	3.5–7	polygonal	depressed	smooth	convex	sparsely present	present	sparse rodlets
15	*E. cavaleriei*	10–18	2.5–7	polygonal	depressed	smooth	flat	present	present	rodlets
16	*E. grandiflora*	10–19	3–5.5	polygonal	depressed	smooth	flat	present	absent	rodlets
17	*E. laoshanica*	20–40	7–12	polygonal	depressed	smooth	flat	absent	present	rodlets
18	*E. japonica*	10–30	3–11	polygonal	depressed	smooth	convex	absent	present	granules
19	*E. prinoides*	10–15	3.5–7.5	polygonal	depressed	smooth	convex	absent	present	granules
20	*E. malipoensis*	20–45	10–15	polygonal	depressed	smooth	convex	present	present	sparse granules
21	*E. seguinii*	3–6	0.5–1.2	polygonal	depressed	smooth	flat	sparsely present	present	granules
22	*E. henryi*	3–11	0.5–2.7	polygonal	raised	smooth	flat	present	present	sparse granules
23	*R. × delacourii*	2–7	0.5–4	polygonal	depressed	smooth	flat	absent	present	rodlets
24	*R. integerrima*	4–7	2–3.5	polygonal	depressed	smooth	flat	present	present	sparse granules
25	*R. indica*	4–8	1.5–4	polygonal	depressed	smooth	flat	absent	present	rodlets
26	*R. ferruginea*	6–15	2.5–5.5	polygonal	depressed	smooth	convex	absent	present	sparse granule
27	*R. jiulongjiangensis*	3–7	0.6–1.2	polygonal	depressed	smooth	convex	absent	absent	granules
28	*R. lanceolata*	3–8	0.5–1.5	polygonal	depressed	smooth	flat	absent	present	rodlets
29	*R. umbellata*	4–10	2–4	polygonal	depressed	smooth	flat	present	present	rodlets
30	*R. umbellata* var. *liukiuensis*	4–11	0.5–1	polygonal	depressed	smooth	flat	present	present	sparse granules
31	*R. wuzhishanensis*	3–6	1.8–3	polygonal	raised	small, straight	convex	present	present	sparse rodlets
32	*R. major*	7–15	4–6	irregular	raised	small, straight	ridge-convex striated	sparsely present	present	sparse granules

LL: leaf length, LW: leaf width, Upper (adaxial) (U), UECSs: epidermal cell shapes, UACWs: anticlinal walls, UCFPs: cuticular folding patterns, UPCWs: periclinal walls, UP: Papillae, UBTs: base of fallen trichomes, and UWDs: epicuticular wax deposits.

**Table 4:** Abaxial quantitative and qualitative variables assessed for study on the micromorphological features of *Eriobotrya* and *Rhaphiolepis* species under scanning electron microscopy.

No.	LECSs	LACWs	LPCWs	LWDs	LBTs	LSDi	LNGTs	LSDe (per µm^2^)	Stomatal Guard Cell
1	polygonal	raised	convex	rodlets	absent	evenly or regularly	absent	300	open, less sunken
2	polygonal	raised	convex	sparse rodlets	present	evenly or regularly	absent	378	open
3	polygonal	raised	convex	absent	absent	evenly or regularly	absent	545	open, less sunken
4	polygonal	raised	convex	absent	present	evenly or regularly	non-glandular	200	open
5	polygonal	depressed	flat	sparse granules	absent	evenly or regularly	absent	534	sunken, sparely open
6	polygonal	depressed	convex	sparse granules	present	evenly or regularly	absent	245	sunken
7	polygonal	depressed	convex	absent	present	evenly or regularly	absent	511	sunken, less open
8	polygonal	depressed	convex	sparsely granules	present	evenly or regularly	absent	466	open
9	polygonal	raised	convex	sparse rodlets and granules	present	evenly or regularly	non-glandular	578	open
10	irregular, long straight-curve	raised	convex	rodlets	present	evenly or regularly	non-glandular	344	open
11	irregular, long straight-curve	raised	convex	rodlets	present	evenly or regularly	absent	522	open
12	polygonal	depressed	flat	sparse granules	present	evenly or regularly	absent	378	open, less sunken
13	polygonal	depressed	flat	absent	present	evenly or regularly	absent	333	sunken
14	polygonal	raised	convex	sparse granules	present	evenly or regularly	non-glandular	278	open
15	polygonal	depressed	convex	sparse granules	absent	evenly or regularly	absent	134	Open, sunken
16	polygonal	depressed	flat	sparse rodlets and granules	present	evenly or regularly	absent	255	open, less sunken
17	polygonal	depressed	flat	rodlets	present	evenly or regularly	absent	278	sunken, less open
18	polygonal	depressed	convex	rodlets	present	evenly or regularly	non-glandular	189	open
19	irregular, long straight-curve	raised	convex	absent	present	evenly or regularly	non-glandular	267	open
20	polygonal	depressed	convex	sparse granules	present	evenly or regularly	non-glandular	255	sunken, less open
21	polygonal	depressed	flat	sparse granules	present	evenly or regularly	absent	288	open, less sunken
22	polygonal	depressed	flat	rodlets	present	evenly or regularly	absent	389	open
23	polygonal-undulate	raised	convex	rodlets	present	unevenly	absent	344	open
24	irregular, small straight-curve	raised	convex	sparse rodlets and granules	present	unevenly	absent	378	open, less sunken
25	irregular, small straight-curve	raised	convex	rodlets	present	unevenly	absent	334	open
26	irregular, small straight-curve	raised	convex	granules	present	unevenly	non-glandular	455	open
27	irregular, small straight-curve	raised	convex	sparse granules	absent	unevenly	absent	378	open
28	irregular, small straight-curve	raised	convex	rodlets	absent	unevenly	absent	334	open
29	irregular, small straight-curve	raised	convex	absent	present	unevenly	non-glandular	467	open, less sunken
30	irregular, small straight-curve	raised	convex	rodlets	present	unevenly	absent	466	sunken, less open
31	irregular, small straight-curve	raised	convex	sparse granules	present	unevenly	absent	355	open
32	polygonal-undulate or irregular undulate	raised	convex	absent	absent	unevenly	absent	411	open

Abaxial or lower (L), LECSs: Epidermal cell shapes, LACWs: anticlinal walls, LPCWs: periclinal walls, LWDs: epicuticular wax deposits, LBTs: base of fallen trichomes, LSDi: stomatal density, LNGTs: persistent non-glandular trichomes (indumentum), LSDe: number of stomata per µm^2^.

**Table 5:** Summary of the quantitative variables for assessment of the relationships among *Eriobotrya* and *Rhaphiolepis* species under SEM.

No.	PSL (µm)	PSW (µm)	PSOLL (µm)	PSOLW (µm)	SSL (µm)	SSW (µm)	SSE (µm)	SSOLL (µm)	SSOLW (µm)
1	26.56 ± 0.51	18.19 ± 0.25	24.65 ± 0.105	15.002 ± 0.13	21.69 ± 0.767	16.79 ± 1.02	1.32 ± 0.15	18.97 ± 0.79	13.91 ± 0.75
2	30.308 ± 0.31	23.06 ± 0.26	24.394 ± 0.30	16.36 ± 0.53	21.44 ± 1.90	17.74 ± 1.31	1.82 ± 0.23	17.37 ± 0.86	13.25 ± 0.97
3	25.42 ± 0.06	17.95 ± 0.08	22.28 ± 0.24	13.78 ± 0.16	20.33 ± 0.94	17.31 ± 1.15	1.47 ± 0.10	16.42 ± 1.51	13.39 ± 1.72
4	35.08 ± 0.25	23.264 ± 0.17	29.33 ± 0.56	16.72 ± 0.37	26.06 ± 1.71	15.21 ± 2.49	2.71 ± 0.42	22.16 ± 1.86	14.33 ± 1.12
5	30.48 ± 0.18	19.29 ± 0.17	28.21 ± 0.13	14.99 ± 0.17	17.09 ± 0.81	13.39 ± 0.86	1.37 ± 0.14	13.12 ± 0.99	10.06 ± 0.75
6	24.88 ± 0.37	15.87 ± 0.20	20.05 ± 0.49	12.81 ± 0.28	23.41 ± 2.13	18.79 ± 1.57	2.36 ± 0.26	18.35 ± 2.50	13.09 ± 1.08
7	24.66 ± 1.55	15.54 ± 0.57	22.22 ± 1.41	11.42 ± 1.05	18.43 ± 1.58	14.28 ± 1.88	1.42 ± 0.51	15.14 ± 1.44	10.55 ± 0.35
8	Nil	Nil	Nil	Nil	26.7 ± 2.41	20.93 ± 2.77	2.37 ± 0.45	22.79 ± 2.08	15.93 ± 1.72
9	31.02 ± 6.85	20.04 ± 1.86	23.16 ± 0.49	17.11 ± 1.55	22.99 ± 0.79	16.44 ± 0.044	1.71 ± 0.23	19.07 ± 0.78	12.73 ± 0.44
10	28.36 ± 0.95	26.64 ± 0.85	24.15 ± 0.13	20.55 ± 0.67	22.95 ± 1.59	19.74 ± 1.45	2.01 ± 0.25	18.99 ± 1.57	15.55 ± 1.89
11	27.65 ± 0.48	20.56 ± 0.40	23.76 ± 0.11	15.49 ± 0.41	21.84 ± 4.26	16.39 ± 2.83	1.62 ± 0.08	18.25 ± 4.66	12.71 ± 3.38
12	25.79 ± 0.66	19.38 ± 0.35	23.06 ± 0.73	16.02 ± 0.83	21.1 ± 0.89	14.3 ± 0.77	1.31 ± 0.13	17.82 ± 0.83	11.55 ± 0.44
13	29.55 ± 0.49	18.55 ± 0.43	25.18 ± 0.38	15.78 ± 0.12	19.04 ± 5.15	16.38 ± 4.52	1.89 ± 0.11	13.93 ± 2.55	11.89 ± 3.30
14	26.946 ± 0.26	21.85 ± 1.08	22.55 ± 0.13	17.05 ± 1.94	23.24 ± 2.04	19.83 ± 2.34	1.62 ± 0.24	19.09 ± 2.20	16.04 ± 2.34
15	34.19 ± 0.25	17.7 ± 0.19	26.07 ± 3.33	9.49 ± 0.49	25.97 ± 1.14	17.75 ± 0.13	1.91 ± 0.23	22.57 ± 2.83	13.95 ± 0.13
16	Nil	Nil	Nil	Nil	20.75 ± 1.04	16.35 ± 1.17	1.56 ± 0.39	18.99 ± 0.63	11.96 ± 1.01
17	Nil	Nil	Nil	Nil	25.21 ± 1.25	18.98 ± 1.93	2.43 ± 0.28	20.67 ± 0.98	13.68 ± 1.67
18	26.01 ± 1.14	18.98 ± 0.15	22.86 ± 1.94	15.55 ± 0.71	20.36 ± 0.47	15.52 ± 0.16	1.72 ± 0.24	16.98 ± 0.54	12.84 ± 0.19
19	30.21 ± 1.28	21.81 ± 2.71	23.41 ± 0.23	14.91 ± 2.49	26.84 ± 1.92	18.76 ± 3.12	2.48 ± 1.05	21.24 ± 1.28	12.72 ± 1.87
20	22.95 ± 1.52	15.95 ± 0.68	20.39 ± 1.52	13.54 ± 0.92	21.15 ± 2.35	13.91 ± 1.75	1.09 ± 0.17	18.76 ± 2.35	11.21 ± 1.48
21	Nil	Nil	Nil	Nil	20.45 ± 1.89	16.99 ± 1.73	2.033 ± 0.26	15.21 ± 2.23	12.64 ± 1.94
22	29.35 ± 1.33	24.04 ± 0.64	24.65 ± 1.47	20.16 ± 0.65	25.64 ± 1.25	18.32 ± 1.13	1.36 ± 0.178	21.73 ± 1.50	14.81 ± 1.25
23	38.21 ± 1.22	28.62 ± 3.34	30.67 ± 0.51	22.11 ± 2.54	35.07 ± 3.79	28.81 ± 1.11	3.4 ± 0.57	29.06 ± 2.81	22.13 ± 1.27
24	34.26 ± 5.13	29.1 ± 0.57	29.98 ± 4.23	22.51 ± 1.23	31.21 ± 2.88	26.27 ± 1.14	3.6 ± 0.31	24.09 ± 2.96	18.66 ± 1.24
25	40.48 ± 3.32	37.03 ± 2.63	34.31 ± 2.09	21.12 ± 2.28	35.03 ± 1.40	29.22 ± 1.04	3.71 ± 0.48	28.70 ± 1.97	21.12 ± 0.54
26	29.93 ± 4.49	28.29 ± 1.11	27.81 ± 2.06	22.57 ± 1.51	29.3 ± 0.89	25.01 ± 1.97	2.89 ± 0.36	23.72 ± 0.97	19.5 ± 0.76
27	32.04 ± 2.87	24.51 ± 4.13	25.96 ± 2.08	18.36 ± 5.25	29.62 ± 1.15	23.87 ± 1.11	3.95 ± 0.22	22.84 ± 1.62	16.21 ± 1.03
28	39.09 ± 1.19	31.18 ± 2.49	32.98 ± 0.17	22.25 ± 4.25	34.93 ± 1.65	28.53 ± 1.48	3.14 ± 0.37	29.11 ± 1.31	21.98 ± 1.22
29	33.22 ± 0.76	28.27 ± 1.31	26.08 ± 1.45	20.91 ± 1.48	31.93 ± 0.26	25.95 ± 0.47	3.65 ± 0.49	25.18 ± 0.49	17.66 ± 1.08
30	32.28 ± 0.75	25.004 ± 4.25	26.96 ± 3.13	19.35 ± 3.34	31.16 ± 1.76	26.11 ± 0.62	3.51 ± 0.21	24.51 ± 0.68	19.25 ± 1.13
31	41.3 ± 5.54	30.49 ± 2.99	36.75 ± 4.32	26.29 ± 2.44	32.09 ± 1.34	30.94 ± 1.14	3.69 ± 0.55	26.38 ± 1.06	23.64 ± 1.91
32	38.86 ± 3.44	31.68 ± 1.48	33.15 ± 3.87	24.42 ± 1.97	29.87 ± 1.75	25.02 ± 1.22	2.62 ± 0.44	24.94 ± 2.22	19.69 ± 0.94

Values are means ± SE. PSL: primary stomata length, PSW: primary stomata width, PSOLL: primary stomata length of outer ledge aperture, PSOLW: primary stomata width of outer ledge aperture, SSL: secondary stomata length, SSW: secondary stomata width, SSE: width of ridge rim, SSOLL: secondary stomata length of outer ledge aperture, and SSOLW: secondary stomata width of outer ledge aperture (SSOLW), Nil indicate data not available.

## Data Availability

All data generated and analyzed during this study are included in the main text and Supplementary Materials. All nuclear and mitochondrial data used in this study can be found in GenBank (accession numbers are provided in Table S2).

## Supplementary Materials

The following supporting information can be downloaded at https://www.mdpi.com/article/10.3390/biology14121740/s1: Figure S1: Mitochondrial DNA (mtDNA) gene tree-based filtered super-networks. (A) All species were included. (B) Four *Eriobotrya* species were excluded. Figure S2: Leaf micromorphology of *Eriobotrya* species (1a–4a indicates adaxial; 1b–4b indicates an abaxial surface). (1) *E. bengalensis*, (2) *E. bengalensis* f. *angustifolia*, (3) *E. condaoensis*, and (4) *E. deflexa*. Scale bars: 50–100 µm. Figure S3: Leaf micromorphology of *Eriobotrya* species (1a–4a indicates an adaxial surface; 1b–4b indicates an abaxial surface). (5) *E. elliptica*, (6) *E. fragrans*, (7) *E. petiolata*, and (8) *E. hookeriana*. Scale bars: 50–200 µm. Figure S4: Leaf micromorphology of *Eriobotrya* species (1a–4a indicates an adaxial surface; 1b–4b indicates an abaxial surface). (9) *E. salwinensis*, (10) *E. tengyuehensis*, (11) *E. obovata*, and (12) *E. serrata*. Scale bars: 50–300 µm. Figure S5: Leaf micromorphology of *Eriobotrya* species (1a–4a indicates an adaxial surface; 1b–4b indicates an abaxial surface). (13) *E. crassifolia*, (14) *E. × daduheensis*, (15) *E. cavaleriei*, (16) *E. grandiflora*, and (17) *E. laoshanica*. Scale bars: 50–300 µm. Figure S6: Leaf micromorphology of *Eriobotrya* species (1a–4a indicates an adaxial surface; 1b–4b indicates an abaxial surface). (18) *E. japonica*, (19) *E. prinoides*, (20) *E. malipoensis*, (21) *E. seguinii*, and (22) *E. henryi*. Scale bars: 50–300 µm. Figure S7: Leaf micromorphology of *Rhaphiolepis* species (1a–4a indicates an adaxial surface; 1b–4b indicates an abaxial surface). (23) *R. × delacourii*, (24) *R. integerrima*, (25) *R. indica*, (26) *R. ferruginea*, and (27) *R. jiulongjiangensis*. Scale bars: 50–300 µm. Figure S8: Leaf micromorphology of *Rhaphiolepis* species (1a–4a indicates an adaxial surface; 1b–4b indicates an abaxial surface). (28) *R. lanceolata*, (29) *R. umbellata*, (30) *R. umbellata* var. *liukiuensis*, (31) *R. wuzhishanensis*, and (32) *R. major*. Scale bars: 50–100 µm. Figure S9: Anatomical description of petiole cross-section outline of *Eriobotrya* and *Rhaphioelpis* species. (1) *E. prinoides*, (2) *E. bengalensis*, (3) *E. japonica*, (4) *R. major*, (5) *R. lanceolata*, and (6) *R. indica*. Scale bars: 2 mm and 500 µm. Figure S10: Micromorphology of fruit surfaces of *Eriobotrya* species. Fruit surface: (1) *E. × daduheensis*, (2) *E. hookeriana*, (3) *E. prinoides*, (4) *E. bengalensis* f. *angustifolia*, (5) *E. japonica*, (6) *E. kwangsiensis*, (7) *E. henryi*, and (8) *E. seguinii*; The outer and inner surfaces of the fruit apical sepals: (9a–9b) *E. crassifolia*, (10a–10b) *E. kwangsiensis*, (11a–11b) *E. deflexa*, and (12a–12b) *E. seguinii*. Designations: The yellow arrows are non-glandular trichomes; the pink arrows are stomata. Scale bars: 1 mm and 200–500 µm. Figure S11: Micromorphology of fruit surfaces of *Rhaphiolepis* species. (1) *R. jiulongjiangensis*, (2) *R. lanceolata*, (3) *R. philippinensis*, and (4) *R. salcifolia*. Designation: The yellow arrows are non-glandular trichomes. Scale bars: 1 mm and 500 µm. Figure S12: Cladogram, Principal Component Analysis (PCA) and Correlation matrix based on 9 stomatal variables between *Eriobotrya* and *Rhaphioelpis*. (A) Cladogram, (B) PCA graph showing contribution of these variables’ attributes to explaining variations in leaf micromorphological features within 32 *Eriobotrya* and *Rhaphiolepis* species. (C) Correlation matrix with *p* < 0.05 significance. Figure S13: Morphological variables of *Eriobotrya* and *Rhaphioelpis*. (1) *Eriobotrya japonica*, (2) *E. prinoides*, (3) *E. bengalensis*, (4) *E. serrata*, (5) *E. henryi*, (6) *E. deflexa*, (7) *E. fragrans*, (8) *E. malipoensis*, (9) *Rhaphiolepis indica*, (10) *R. jiulongjiangensis*, (11) *R. salicifolia*, (12) *R. lanceolata*, (13) *R. ferrugenia*, (14) *R. × delacourii*, (15) *R. umbellata*, and (16) *R. wuzhishanensis*. Scale bars: 50 µm. Table S1: Summary of studied samples included in this study. Number in parentheses indicates the estimated total number of species described in each genus. Number near parentheses indicates the sampling in this study. Table S2: Information on all species included in this study. Table S3: Species and sequence information from NCBI used to construct nuclear phylogeny in this study. Table S4: Micromorphological variables of *Eriobotrya* and *Rhaphiolepis*. Table S5: Individual mitochondrial genes, nucleotide diversity, and GC contents in this study. Table S6: Eigenvalues and cumulative variances for 2 major factors obtained from PCA analysis and 26 variables for each factor within *Eriobotrya* and *Rhaphiolepis*. Table S7: Statistical analysis of 9 quantitative stomatal variables within *Eriobotrya* and *Rhaphiolepis* species. Table S8: Eigenvalues and cumulative variances for 2 major factors obtained from PCA analysis and 9 quantitative stomatal variables for each factor within *Eriobotrya* and *Rhaphiolepis*.

## Abbreviations

The following abbreviations are used in this manuscript:

nrDNA	Nuclear Ribosomal DNA
mtDNA	Mitochondrial DNA
SEM	Scanning Electron Microscope
PCA	Principal Component Analysis
