# Supplementary figures and images for: Reassessing the Evolutionary Relationships of Eriobotrya and Rhaphiolepis (Rosaceae): Evidence from Micromorphology, Complete Nuclear Ribosomal DNA and Mitochondrial Genomic Data
Source: Biology (Basel). 2025 Dec 4;14(12):1740. doi: 10.3390/biology14121740 (PMC12731197; doi:10.3390/biology14121740)

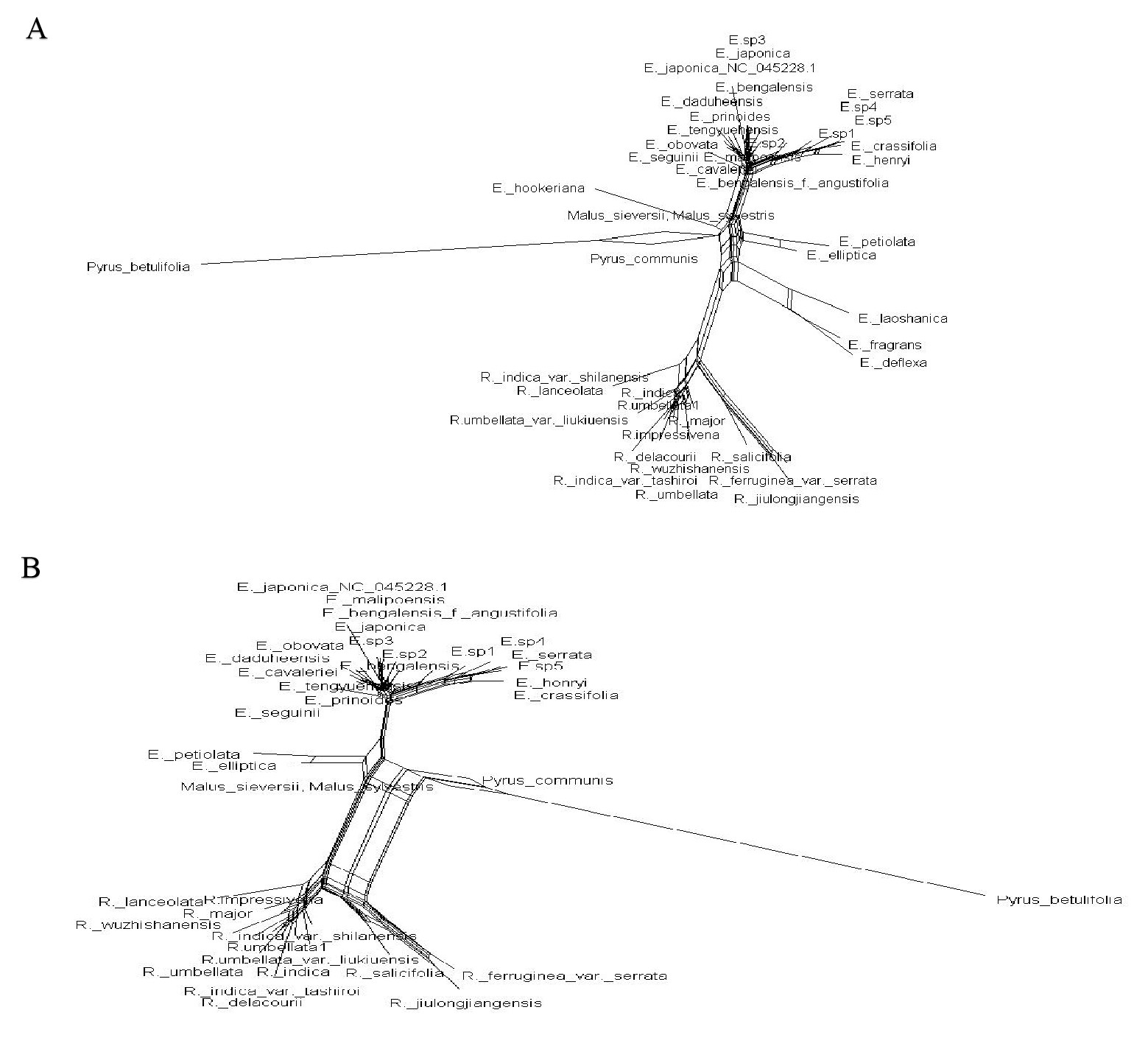

Supplement: Supplementary file 1 [file biology-14-01740-s001.zip › Figure S1..jpg]

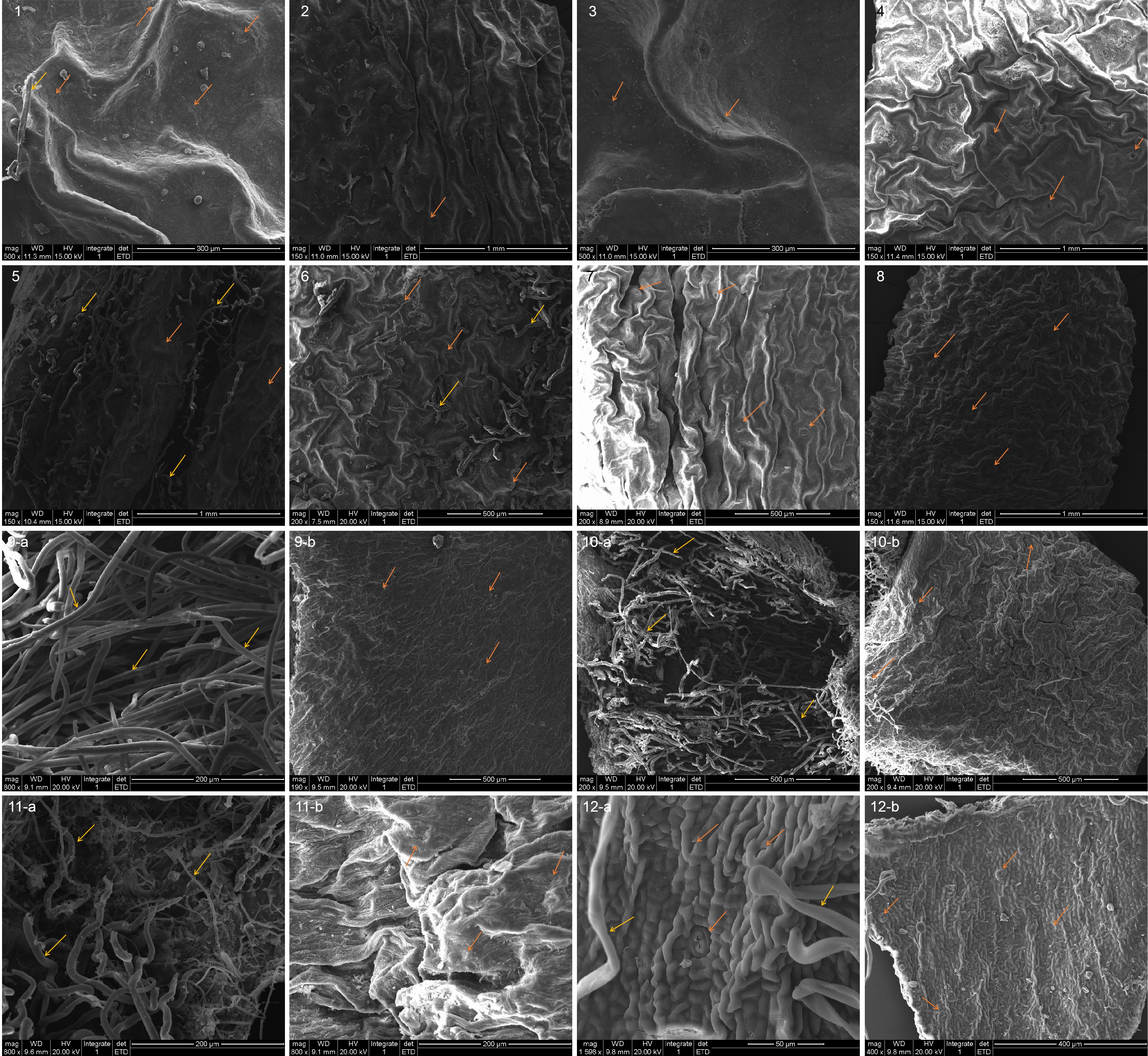

Supplement: Supplementary file 1 [file biology-14-01740-s001.zip › Figure S10..jpg]

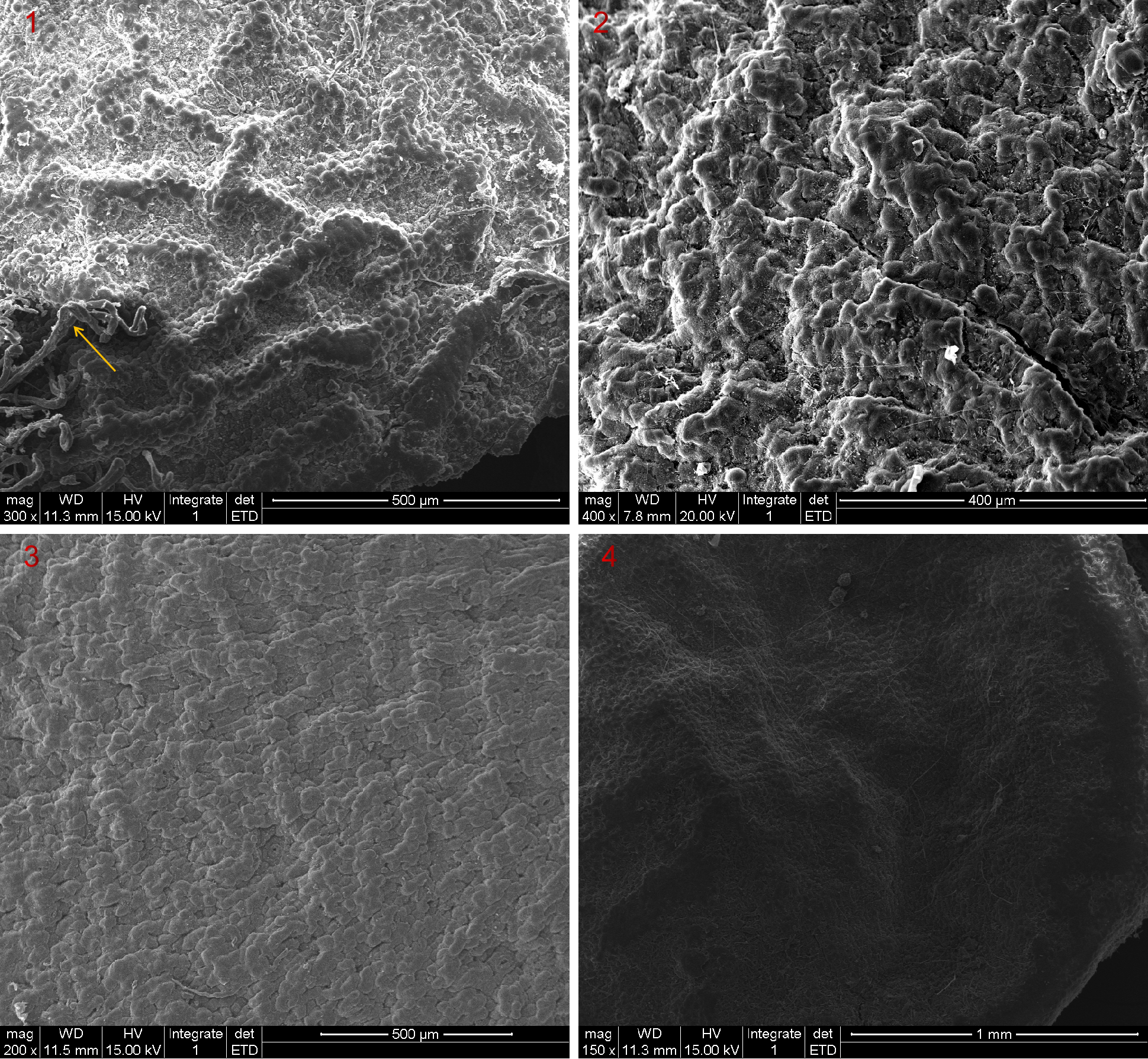

Supplement: Supplementary file 1 [file biology-14-01740-s001.zip › Figure S11..png]

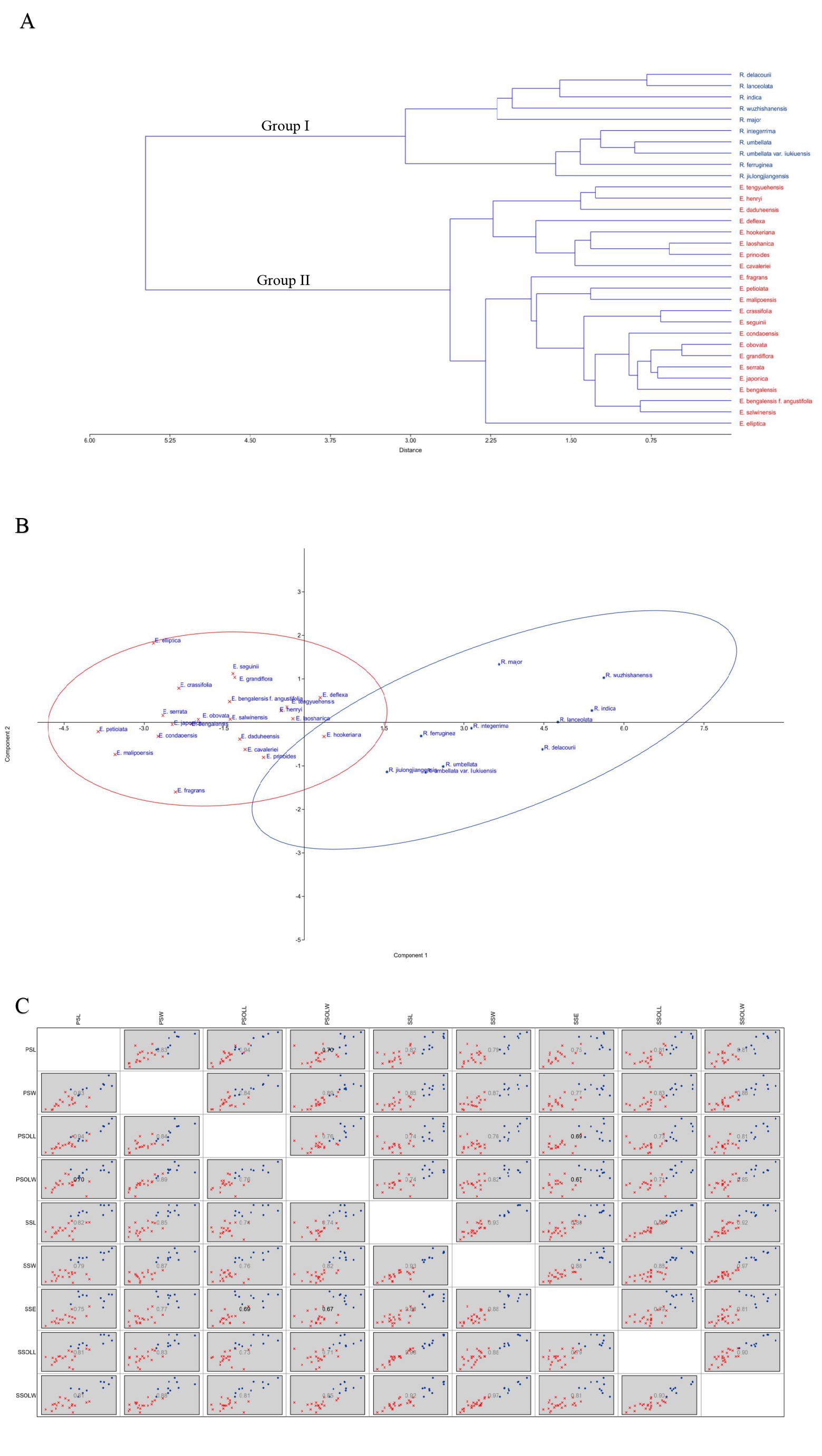

Supplement: Supplementary file 1 [file biology-14-01740-s001.zip › Figure S12..jpg]

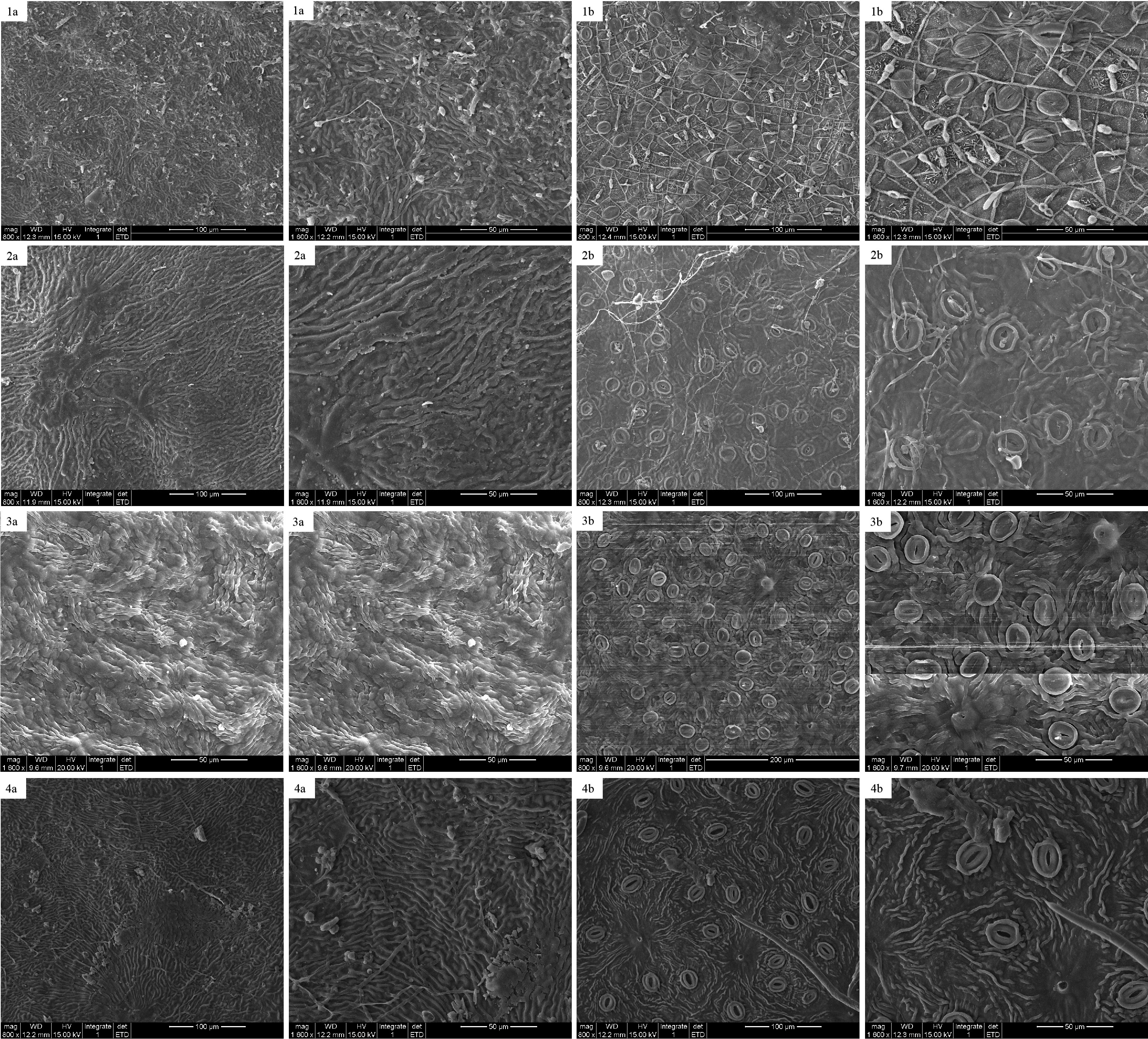

Supplement: Supplementary file 1 [file biology-14-01740-s001.zip › Figure S2..jpg]

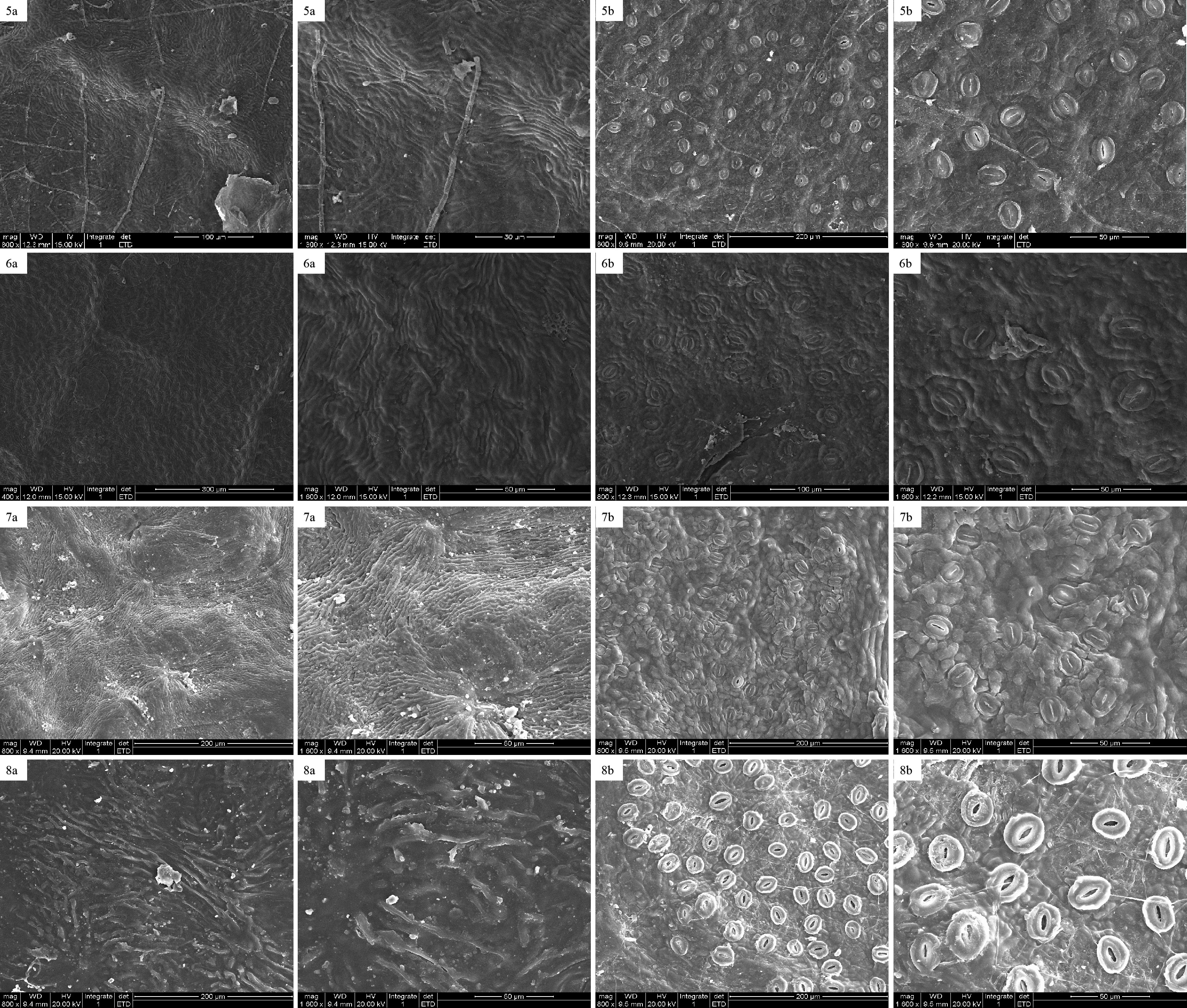

Supplement: Supplementary file 1 [file biology-14-01740-s001.zip › Figure S3..jpg]

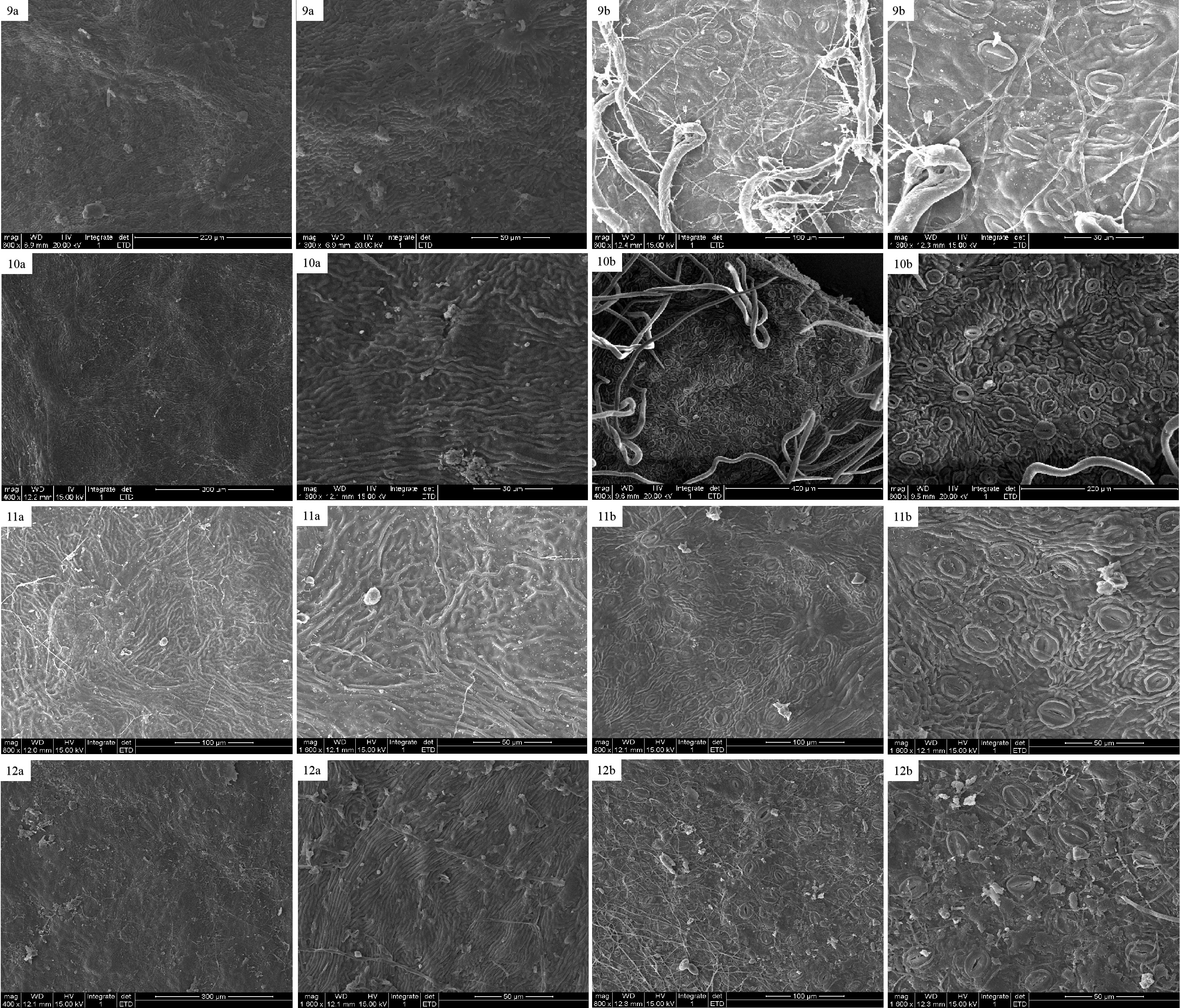

Supplement: Supplementary file 1 [file biology-14-01740-s001.zip › Figure S4..jpg]

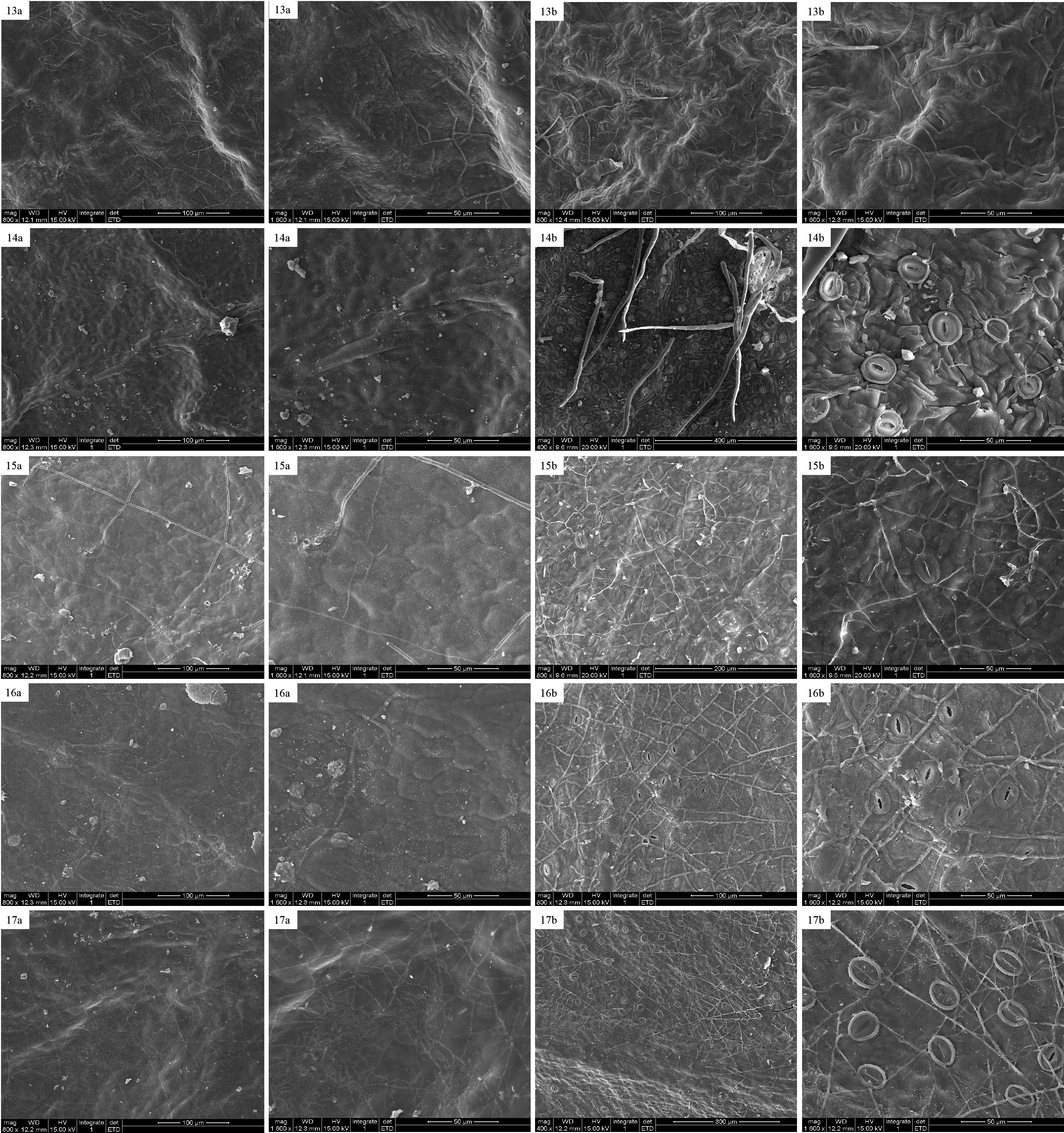

Supplement: Supplementary file 1 [file biology-14-01740-s001.zip › Figure S5..jpg]

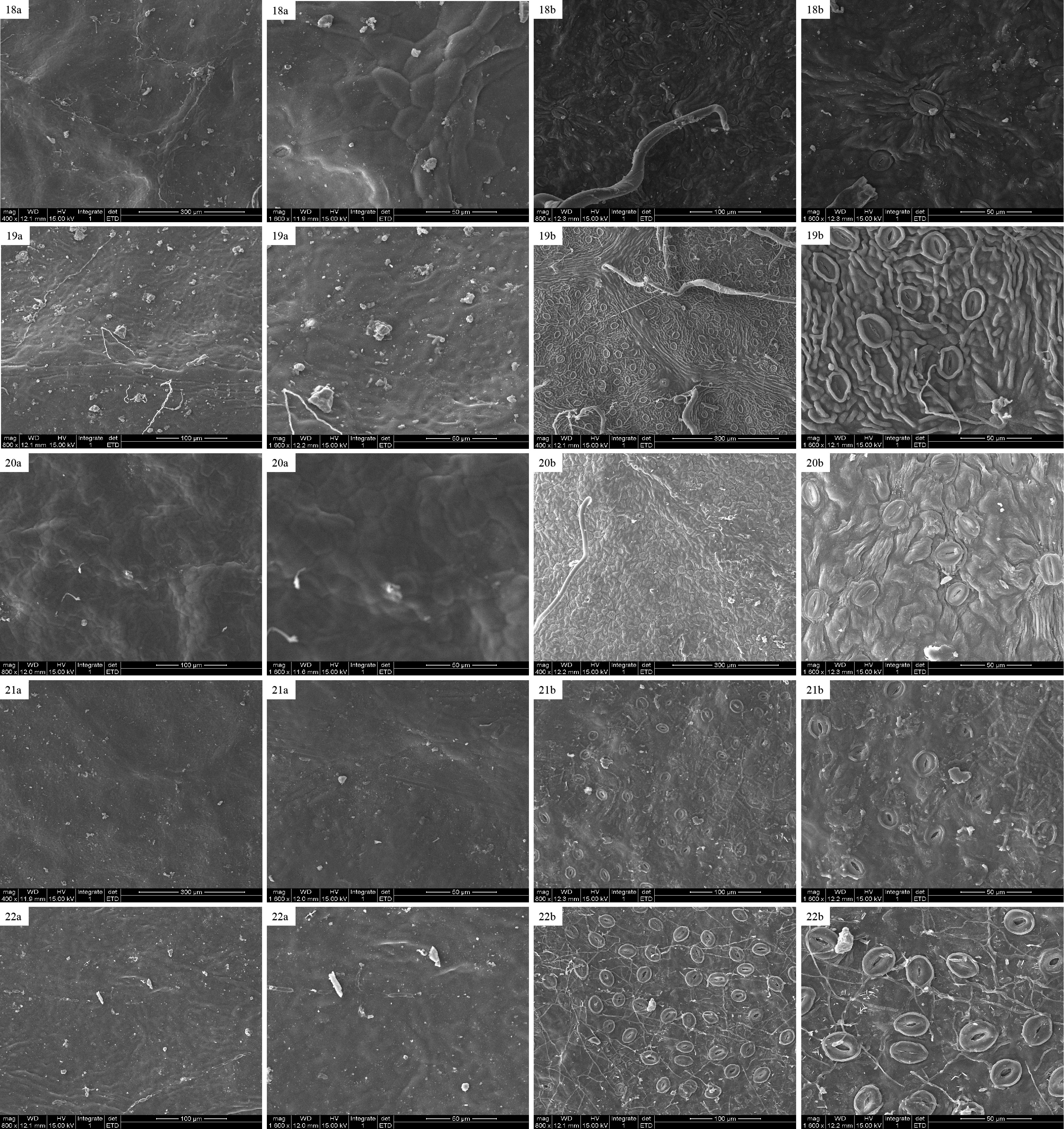

Supplement: Supplementary file 1 [file biology-14-01740-s001.zip › Figure S6..jpg]

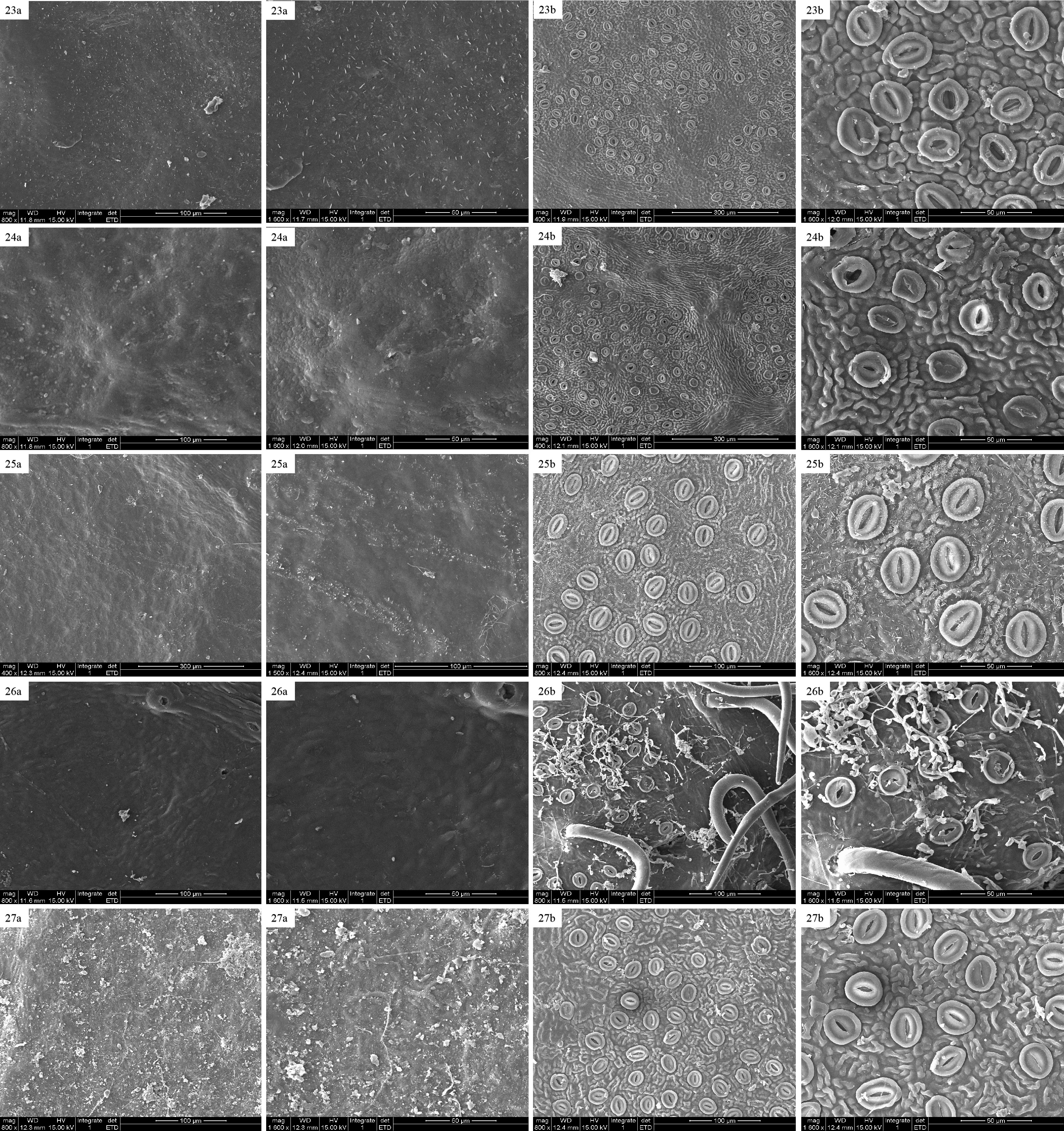

Supplement: Supplementary file 1 [file biology-14-01740-s001.zip › Figure S7..jpg]

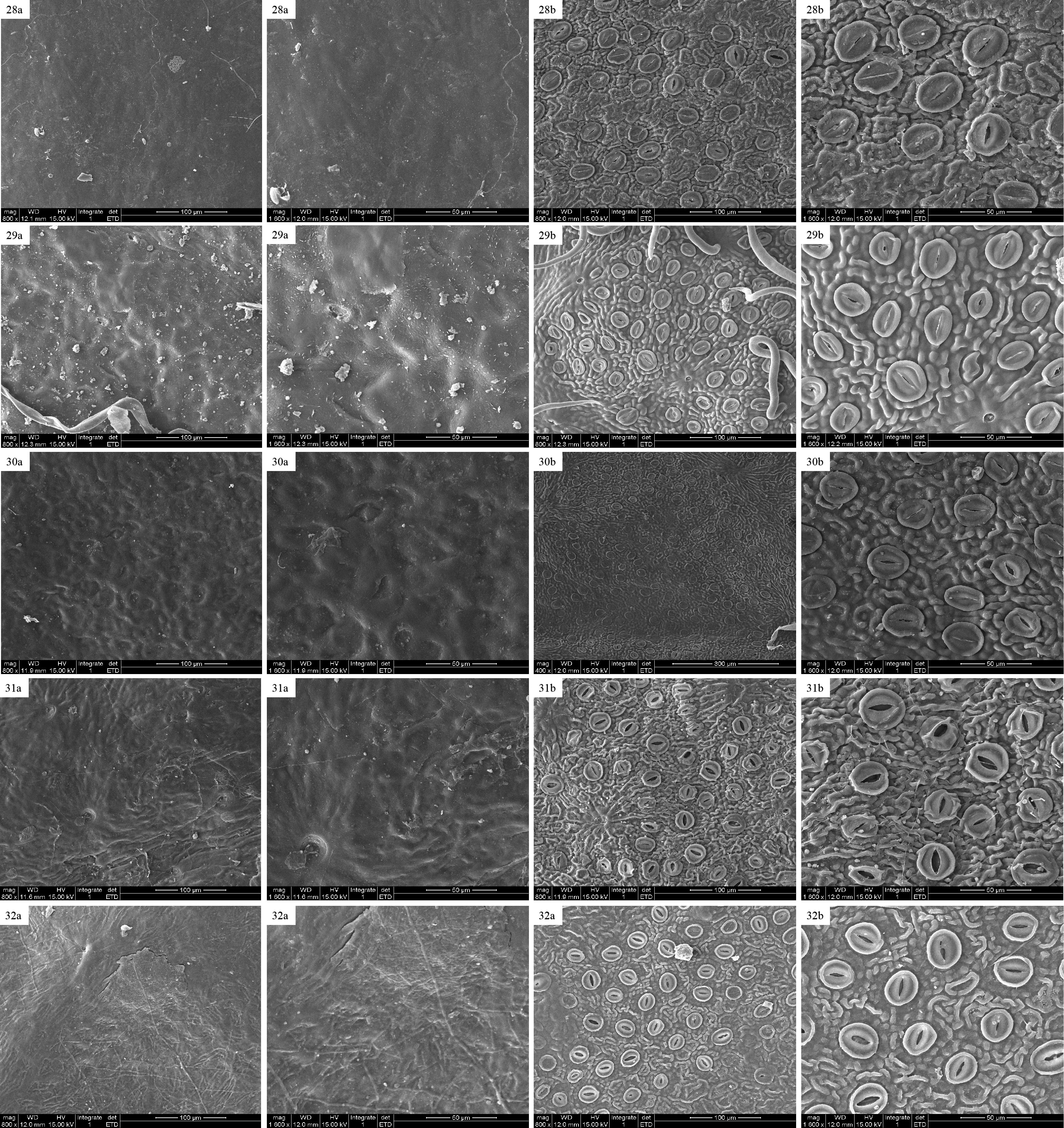

Supplement: Supplementary file 1 [file biology-14-01740-s001.zip › Figure S8..jpg]

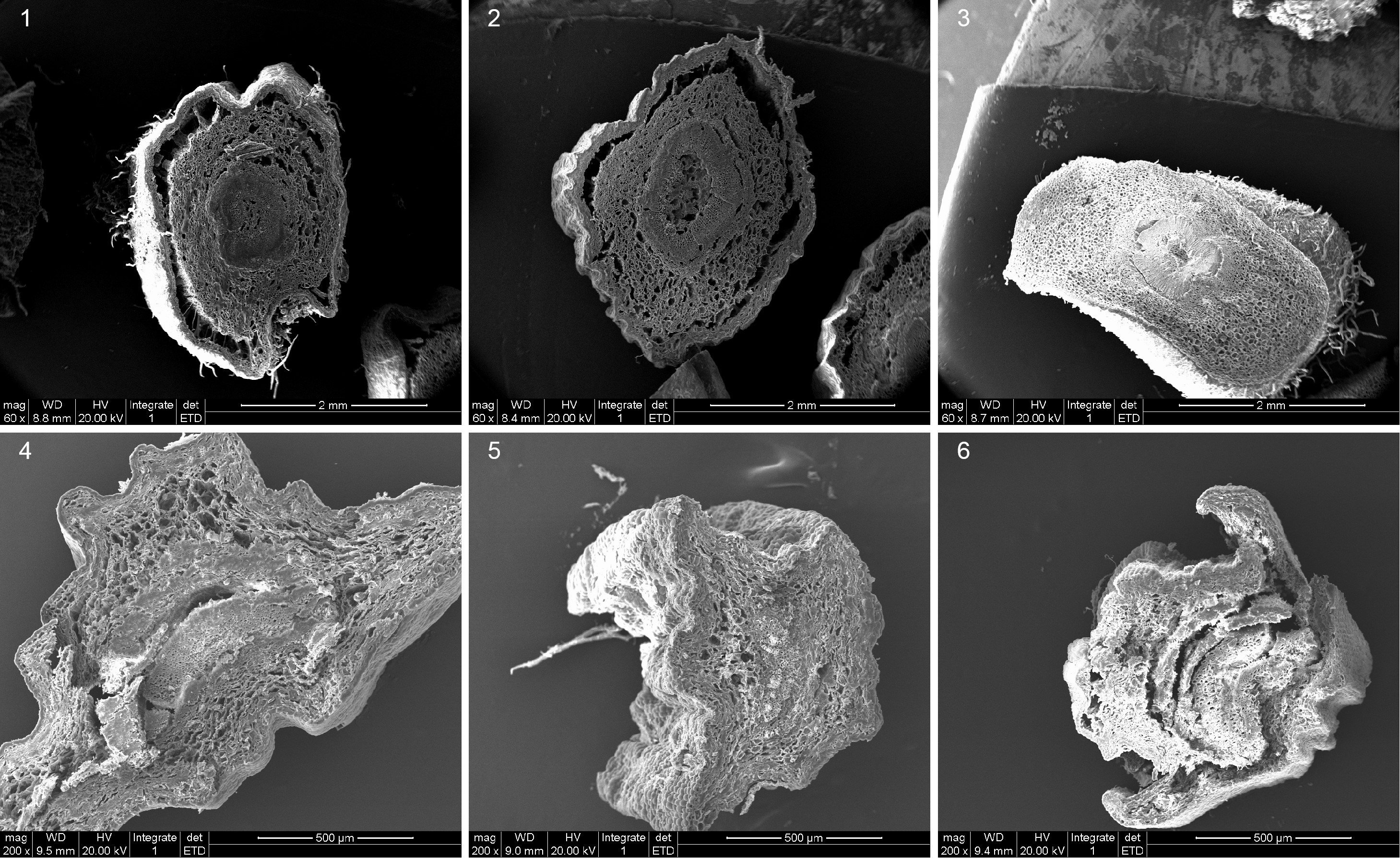

Supplement: Supplementary file 1 [file biology-14-01740-s001.zip › Figure S9..jpg]
